# An integrated nitrogen utilization gene network and transcriptome analysis reveal candidate genes in response to nitrogen deficiency in *Brassica napus*

**DOI:** 10.3389/fpls.2023.1187552

**Published:** 2023-05-09

**Authors:** Pengfeng Li, Runjie Du, Zhaopeng Li, Zhuo Chen, Jiana Li, Hai Du

**Affiliations:** ^1^ College of Agronomy and Biotechnology, Chongqing Engineering Research Center for Rapeseed, Southwest University, Chongqing, China; ^2^ Academy of Agricultural Sciences, Southwest University, Chongqing, China

**Keywords:** *Brassica napus*, nitrogen, genes, evolution, expression, RNA-seq

## Abstract

Nitrogen (N) is an essential factor for crop yield. Here, we characterized 605 genes from 25 gene families that form the complex gene networks of N utilization pathway in *Brassica napus*. We found unequal gene distribution between the A_n_- and C_n_-sub-genomes, and that genes derived from *Brassica rapa* were more retained. Transcriptome analysis indicated that N utilization pathway gene activity shifted in a spatio-temporal manner in *B. napus*. A low N (LN) stress RNA-seq of *B. napus* seedling leaves and roots was generated, which proved that most N utilization related genes were sensitive to LN stress, thereby forming co-expression network modules. Nine candidate genes in N utilization pathway were confirmed to be significantly induced under N deficiency conditions in *B. napus* roots, indicating their potential roles in LN stress response process. Analyses of 22 representative species confirmed that the N utilization gene networks were widely present in plants ranging from Chlorophyta to angiosperms with a rapid expansion trend. Consistent with *B. napus*, the genes in this pathway commonly showed a wide and conserved expression profile in response to N stress in other plants. The network, genes, and gene-regulatory modules identified here represent resources that may enhance the N utilization efficiency or the LN tolerance of *B. napus*.

## Introduction

Nitrogen (N) is a vital component of many important plant compounds (e.g., nucleotides, amino acids, and chlorophyll), and plants obtain it from the soil in inorganic or organic forms (Kiba and Krapp, 2016). N acquisition, which affects photosynthesis, metabolism, growth, and crop yield, is essential for plant growth and development (Wang et al., 2014). N fertilizers are extensively applied to ensure maximum crop yields (Ju et al., 2009), however, only 30-40% of applied N fertilizer is used by plants, while the majority is lost, leading to a series of complications associated with resource wastage, environmental pollution, etc. To address these challenges and promote sustainable agriculture, it is important to develop crops that are more tolerant to low nitrogen (LN) conditions, which would reduce the need for N fertilizers under varying N conditions. A proper understanding of the molecular basis of N utilization is vital for achieving this goal.

The N utilization process mainly includes the N uptake, transport and assimilation, which is coordinately regulated by a set of structural genes and regulatory genes in plants. Firstly, N (e.g., nitrate) is taken up by the roots through specific transporters such as NRT1.1 (CHL1/NPF6.3)/NRT1.5/1.6 (Tsay et al., 1993; Almagro et al., 2008; Lin et al., 2008) and NRT2.1/2.2/2.4 (Cerezo et al., 2001; Filleur et al., 2001). After being transported into plant cells, nitrate is first reduced to nitrite and then to ammonium by nitrate reductase (NR) and nitrite reductase (NiR) respectively, following which ammonium is assimilated into amino acids *via* glutamine synthetase (GS)/glutamine-2-oxoglutarate aminotransferase (GOGAT) cycle (Ali et al., 2008; Kissen et al., 2010; Lothier et al., 2011).

To date, the gene networks related to N utilization have been well understood in model plant *Arabidopsis*. The N uptake network consists of four transmembrane protein families (NRT2, Nitrate transporter 2; NPF, Nitrate transporter 1/Peptide transporter; NAR2, Nitrate reductases; and AMT1, Ammonium transporter 1) (Tsay et al., 1993; Huang et al., 1999; Cerezo et al., 2001; Lupini et al., 2016) and 14 types of regulatory proteins, e.g., CIPK8 (CALCINEURINB-LIKE INTERACTING SER/THR-PROTEINE KINASE8) (Marchive et al., 2013), NLP7 (NIN-LIKE PROTEIN7) (Liu et al., 2017). The N transport network includes members of five transmembrane protein families (NPF; NRT2; AMT2, Ammonium transporter 2; CLC, Chloride channel; and SLAC/SLAH, Slow anion channel-associated homologues) (Sohlenkamp et al., 2002; Chopin et al., 2007; Almagro et al., 2008; Lin et al., 2008; Krapp et al., 2014) and one transcription factor (TF) family (R2R3-MYB, MYB59) (Du et al., 2019). The assimilation network comprises four types of enzymes, namely NR (NIA1 and NIA2 proteins), NiR (NiR1 protein), GS (e.g., GLN1.1 and GS2 proteins), and GOGAT (e.g., GLU1 and GLT1 proteins) (Ali et al., 2008; Kissen et al., 2010; Lothier et al., 2011; Zhao et al., 2016), and eight types of regulatory protein, such as CBL1/9 (Calcineurin B-like proteins), etc (Li et al., 2020; Liu et al., 2021). In addition, some regulatory proteins, such as LBD37/38/39 (LATERAL ORGAN BOUNDARY DOMAIN transcription factors), play a significant role in both N uptake and assimilation networks (Rubin et al., 2009; Liu et al., 2017).

Despite significant progress in characterizing genes involved in N utilization in plants, our current understanding is primarily derived from scattered experimental studies of gene function in various plant species. Except for *Arabidopsis*, the N utilization network in most plants remains poorly understood, and our knowledge of the distribution, origin, and evolutionary history of the N utilization pathway is limited. To overcome these limitations, a comprehensive analysis of gene networks related to N utilization at a genome-wide level could aid in identifying important genes involved in N utilization and LN tolerance, particularly in crops. The increasing number of sequenced plant genomes provides a robust database for systematically identifying N utilization gene networks and exploring the mechanisms underlying their evolution at a genome-wide level.

*B*. *napus* (genome AACC, 2n = 38) is an oil crop found worldwide which was originated *via* hybridization between *Brassica rapa* (genome AA, 2n = 20) and *Brassica oleracea* (genome CC, 2n = 18) approximately 7500 years ago (Chalhoub et al., 2014), and provides a valuable model for exploring the evolution of N utilization pathway following an allopolyploid event in plants. In this study, we first identified and constructed the gene networks of the N utilization pathway in *B. napus*, and explored the regulatory and evolution mechanisms. We also explored the evolutionary history of this pathway in other 19 representative species of algae, tracheophytes and flowering plants. Moreover, to explore the LN stress response mechanism and identify the potential LN responsive genes in *B. napus*, we constructed a transcriptomic dataset of *B. napus* Zhongshuang 11 (ZS11) ecotype exposed to 1, 3, 5, and 12 d of LN stress at the five-leaf stage, using both root and leaf tissue samples. The LN response mechanism in *B. napus*, and the LN stress and spatio-temporal expression profile and co-expression network of this pathway were also investigated based on the RNA-seq data. The genes in this pathway that were significantly differentially expressed under LN stress were screened. These data provide a detailed overview and assessment of the distribution, evolution, and expression patterns of N utilization pathway in plants.

## Materials and methods

### Plant materials

ZS11 seeds were obtained from the College of Agriculture and Biotechnology, Southwest University (Beibei, Chongqing, China). The seeds were sown in soil (nutrition soil and vermiculite mixed in a 2:1 ratio) and grown in an artificial climatic chamber at 22°C with a 16/8 h (day/night) photoperiod. Then, 48 healthy plants were selected for preculture at the three-leaf stage, with each plant was transferred to 250 milliliter Hoagland liquid medium (500 μM K_2_SO_4_, 250 μM KH_2_PO_4_, 325 μM MgSO_4_, 50 μM NaCl, 8 μM H_3_BO_3_, 0.4 μM MnSO_4_, 0.4 μM ZnSO_4_, 0.4 μM CuSO_4_, 0.1 μM Na_2_MoO_4_, 40 μM Fe-EDDHA, 10 μM C_2_H_4_N_4_, 1.8mM Ca(NO_3_)_2_, 0.2mM (NH_4_)_2_SO_4_ and pH=5.8). Seedlings at the five-leaf stage were used for LN treatments in the same volume of adjusted Hoagland liquid medium with changed Ca(NO_3_)_2_ (normal N condition 1.8 mM and N deficiency condition 0.09 mM), and (NH_4_)_2_SO_4_ (normal N condition 0.2 mM and N deficiency condition 0.01 mM) concentration (Koeslin-Findeklee et al., 2015). Root and leaf tissues were collected on days 1, 3, 5, and 12 after treatment and immediately frozen in liquid nitrogen, respectively. Each treatment was replicated twice, and each replicate containing three plants. All samples were stored at -80°C for RNA extraction.

Total RNA was extracted using an EASYspin total RNA Extraction Kit (Biomed, Beijing, China). RNA degradation and contamination were monitored on 1% agarose gels. The quality and concentration of total RNA were examined using gel electrophoresis and a NanoPhot-ometer^®^ spectrophotometer (IMPLEN, CA, USA) (A260/280 ratio = 1.8-2.1; A260/230 ratio ≥ 2.0), respectively.

### RNA sequencing and data analysis

A total of 32 samples were sequenced on an Illumina Hiseq Xten platform at BioMarker (Beijing, China). Each sample set contained two biological replicates. The reads with read length over 150bp were used for subsequent analysis, and the average sequencing depth per sample were more than 6X. Then, FastQC and Trimmomatic software were applied for quantity control with the default parameters. One sample of roots under normal N condition was excluded from the dataset on day 5, due to low sequencing quality. Finally, Hisat2 was used for mapping the genome. The dataset has been submitted to the NCBI (Accession number: PRJNA612634). Differential expression analysis of two conditions/groups was performed using the DESeq R package (1.10.1). The PCA, Venn, GO and KEGG analysis were performed by a toolkit in BMKCloud (www.biocloud.net).

### Identifying N utilization pathway genes

The sequences of 109 known *Arabidopsis* N utilization genes were retrieved from TAIR (https://www.arabidopsis.org/) (**Table S1**). To identify N utilization pathway genes in the *B. napus* genome, a BLASTP search against the proteome sequences of Darmor–*bzh* ecotype in GENOSCOPE database (http://www.genoscope.cns.fr/brassicanapus/) was applied using the protein sequence of each *Arabidopsis* N utilization gene as query respectively. To verify the reliability of the results, candidate sequences were further verified *via* SMART (http://smart.embl-heidelberg.de/) and PFAM (http://pfam.xfam.org/) to ensure they contained the typical domains of each gene family, respectively. Next, MAFFT online software (https://mafft.cbrc.jp/alignment/server/) was used for multiple sequence alignment of all members in each gene family in *B. napus* and *Arabidopsis* with default settings, respectively. Then, a neighbor-joining (NJ) tree of each gene family was constructed based on the multiple sequence alignment using MEGA 7.0 with the following parameters: pairwise deletion, p-distance model, and bootstrap analysis of 1,000 replications (Kumar et al., 2016). Finally, the candidates were considered as positive hits based on the following two criteria: (1) the genes were most closely related to the known *Arabidopsis* N utilization genes in the phylogenetic tree (NJ tree); (2) the genes shared a higher sequence similarity with that of the known *Arabidopsis* N utilization genes. The DNA, CDS and protein sequences of candidates were obtained *via* GENOSCOPE. Subcellular localization was predicted by Cell-PLoc2.0 (http://www.csbio.sjtu.edu.cn/bioinf/Cell-PLoc-2/).

The same procedure and criteria were followed to identify the homologs from another 20 sequenced plant genomes in Phytozome (http://www.Phytozome.net), including green algae (*Volvox carteri*, *Chlamydomonas reinhardtii* and *Klebsormidium nitens*), moss (*Physcomitrella paten*), liverwort (*Marchantia polymorpha*), tracheophytes (*Selaginella moellendorffii*), gymnosperms (*Picea abies*), basal magnoliophyta (*Amborella trichopoda*), monocots (*Zea mays* and *Oryza sativa*), and eudicots (*Aquilegia coerulea*, *Solanum lycopersicum*, *Solanum tuberosum*, *Eucalyptus grandis*, *Vitis vinifera*, *Populus trichocarpa*, *Brassica rapa*, *Brassica oleracea*, *Glycine max*, and *Medicago truncatula*).

### Chromosomal location and synteny analysis of N utilization-related genes

Information on chromosome localization of candidates were obtained from the GENOSCOPE *B. napus* genome database. Collinearity of candidates in *B. oleracea*, *B. rapa* and *B. napus* genomes were analyzed using CoGe online software (https://genomevolution.org/CoGe/). Duplication events were defined based on the cross-genome collinearity analysis of candidates (orthologous gene pairs in orthologous blocks). Tandem duplicated genes were defined as the closely related genes in a single cluster in the phylogenetic tree that were physically localized adjacent to each other on a given chromosome with no more than one gene intervening. The nucleotide substitution rate (Ka/Ks) of candidates was calculated by KaKs_calculator2.0, using the LWL method (Wang et al., 2010).

### Promoter and miRNA regulation networks of N utilization pathway

Potential *cis*-acting regulatory elements in upstream promoter sequences (-1,500 bp) of candidates were predicted using PlantCARE (http://bioinformatics.psb.ugent.be/webtools/plantcare/html/) with default parameters. The transcription factor-binding site was predicted by PlantTFDB database (http://planttfdb.cbi.pku.edu.cn/prediction.php) using -1,500 bp promoter sequences with threshold *P*-value less than 1e-7. Potential regulatory miRNAs of candidates were predicted *via* psRNATarget online software (http://plantgrn.noble.org/psRNATarget/analysis?function=2) using the CDS sequences of candidates with default parameters, respectively.

### Spatio-temporal and LN stress expressions of N utilization pathway

To analyze the spatio-temporal expression profile of candidates in *B. napus*, the RNA-seq dataset of 60 ZS11 samples from seven major organs (roots, hypocotyls, stem, leaves, flowers, silique pericarps, and seeds) at six main development stages (seed germination, seeding, budding, initial flowering, full bloom, and seed maturation) were acquired from NCBI (BioProject ID PRJNA358784). The expression profile of candidates in root and leaf tissues of ZS11 seedlings under LN treatments was obtained from the RNA-seq dataset constructed in this study (BioProject ID PRJNA612634). Any gene with weak (fragments per kilobase of transcript per million fragments mapped reads, FPKM < 1) or no detectable expression levels in all samples was excluded from further analysis. The genes were defined as differentially expressed genes (DEGs) if the FC (Fold Change) ≥ 2. RNA-seq data of candidates were log2-transformed and clustered by Cluster 3.0 (de Hoon et al., 2004), and then visualized using Java Treeview (Saldanha, 2004).

The public N stress RNA-seq datasets of *Arabidopsis*, *S. tuberosum*, *S. lycopersicum*, *O. sativa* and *Z. mays*, with accession numbers GSE97500, PRJNA529319, GSE139405, and GSE107562 respectively, were obtained from the Gene Expression Omnibus (GEO) database in NCBI (https://www.ncbi.nlm.nih.gov/geo/). The *O. sativa* LN and HN stress RNA-seq dataset (Xin et al., 2019a; Xin et al., 2019b) were provided by the authors directly. The expressions of N utilization pathway genes in the corresponding species were analyzed by the same method used in *B. napus*.

### Co-expression analysis of *B. napus* N utilization pathway

The Pearson correlation coefficient (PCC) of candidates was calculated using the R package (*Psych*). The threshold value for co-expressed genes is |PCC value| > 0.6. The co-expression network was visualized by Cytoscape (Shannon et al., 2003). The core gene-network was calculated by the MCODE plugin in Cytoscape with default parameters. The potential hub gene in each gene network was calculated by the cytoHubba plugin with default parameters, and the top ten genes with high degree value were screened.

### qRT-PCR analysis of N utilization-related genes under LN conditions in *B. napus*

The expression patterns of nine putative LN stress responsive genes were analyzed by qRT-PCR method, using *BnActin7* (BnaA02G0033600ZS) and *BnaUBI* (BnaC08T0011500ZS) as double internal controls. The candidate genes and primers used in this analysis were listed in **Table S2**. The plant material, experimental treatment, and total RNA extraction methods were consistent with that of the RNA-seq analysis. Subsequent first-strand cDNA synthesis was performed in a 20 μL reaction system using 1 μg of total RNA according to the manufacturer’s instructions of HiScript^®^ III RT SuperMix for qPCR (+gDNA wiper) HiScript^®^ III RT kit (Vazyme, Nanjing, China). Real-time PCR analysis was performed in a CFX Connect™ Real-Time System (Bio-Rad, USA) by using Taq Pro Universal SYBR qPCR Master Mix Kit (Vazyme, Nanjing, China). The qPCR reaction parameters were as follows: initial denaturation at 95°C for 3 min, followed by 40 cycles of denaturation at 95°C for 10 s, and annealing at 58-60°C for 20 s. The melting curve was measured at 95°C for 10 s, 60°C for 60 s and 95°C for 15 s. The presence of a single peak in the melting curve confirmed the specificity of the primers. Finally, gel electrophoresis was conducted to verify the size of the PCR products. Each treatment included three biological replicates, and each consisted of three technical replicates. The relative gene expression levels of candidates were calculated using the 2(^−ΔΔCt^) method. Difference in expression level of each gene was assessed by One-way ANOVA analyses of variance (*p<0.05; **p<0.01) using Excel 2016.

## Results

### Identifiction and construction of N utilization pathway genes in *B. napus*

**Figure 1:**
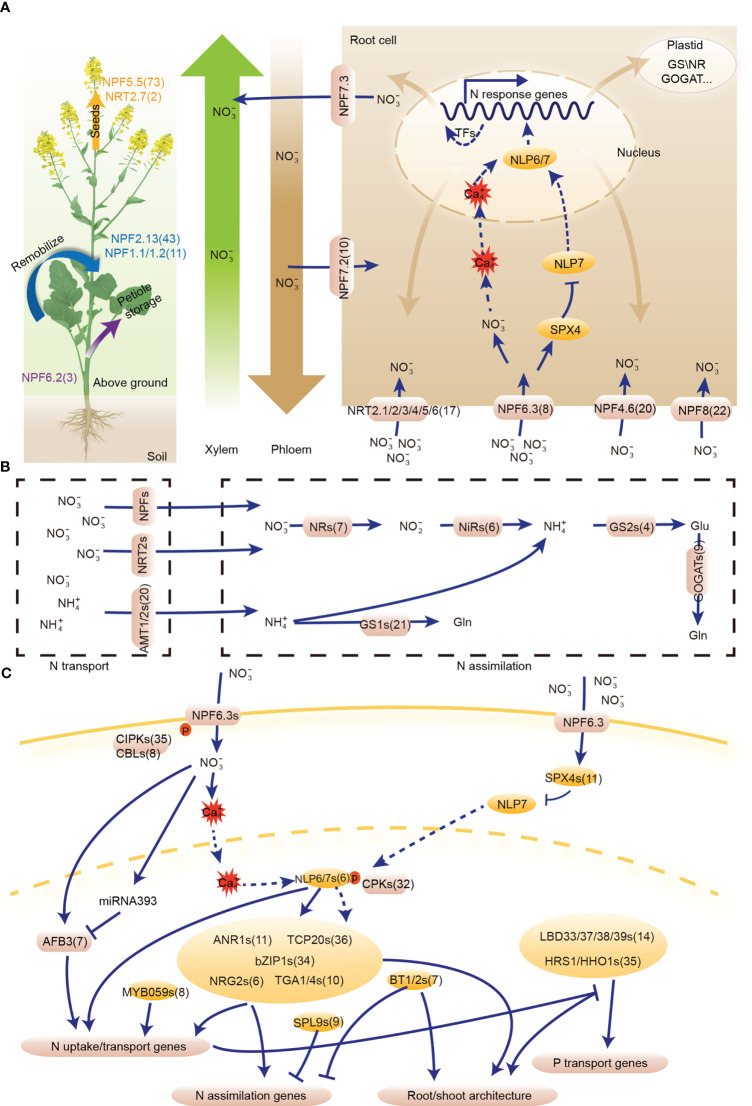
The nitrogen **(N)** utilization pathway in *Brassica napus*. Nitrate is sensed by *NPF6.3*, which transfers the N signal to *NLP6/7 via* phosphorylation. The hub gene, *NLP6/7*, regulates downstream TFs and structural genes to utilize N. Moreover, *NLP6/7* indirectly regulates root morphology under different N conditions. **(A)** N uptake and transport gene network. **(B)** N assimilation gene network. **(C)** Common regulatory genes of N uptake and assimilation gene networks. Transcription factor (TF) is indicated by an oval, while the rounded rectangles represent structural genes and enzymes. The numbers following the gene name indicate the gene number of the homologous gene in *B. napus*. NPF, NITRATE TRANSPORTER 1/PEPTIDE TRANSPORTER; AFB, AUXIN SIGNALING F-BOX PROTEIN; ANR, ARABIDOPSIS NITRATE REGULATED; BT, BTB and TAZ DOMAIN PROTEIN; bZIP, BASIC LEUCINE ZIPPER; CBL, CALCINEURIN B-LIKE PROTEIN; CIPK, CBL-INTERACTION PROTEIN KINASE; CPK, CALCIUM-SENSOR PROTEIN KINASE; HHO, HRS1 HOMOLOG; HRS, HYPERSENSITIVITY TO LOW PI-ELICITED PRIMARY ROOT SHORTENING; LBD, LATERAL BOUNDARY DOMAIN-CONTAINING PROTEIN; NAC, NAM-ATAF-CCUC DOMAIN-CONTAINING PROTEIN; NLP, NIN-LIKE PROTEIN; NRG, NITRATE REGULATORY GENE; NRT, NITRATE TRANSPORTER; SPL, SQUAMOSA PROMOTER BINDINGPROTEIN-LIKE; TCP, TEOSINTE BRANCHED1/CYCLOIDEA/PROLIFERATING CELL FACTOR; TGA, TGACG MOTIF-BINDING FACTOR; NR, Nitrate reductase; NiR, Nitrite reductase; GS, Glutamine synthetase; GOGAT, Glutamine-2-oxoglutarate aminotransferase.

We identified 605 N utilization pathway genes from 25 gene families in the *B. napus* genome, including 201 genes involved in N uptake, 206 in N transport, 56 in N assimilation networks, and 142 common regulatory genes involved in N uptake and assimilation networks (**Table S1**). In N uptake network, 41 of the 201 candidates were homologs from NRT2 (17 genes), NAR2 (10 genes) and AMT1 (14 genes) high-affinity gene families, 50 were homologs from low-affinity NPF family, while 110 were homologs of regulatory genes, including *AFB3* (*Abscisic Acid Responsive Element Binding Factor 3*, seven genes), *ANR1* (*Nitrate regulated 1*, 11 genes), *NRG2* (*NITRATE REGULATORY GENE2*, six genes), *bZIP1* (*basic leucine zipper domain 1*, 34 genes); *HRS1/HHO1* (*HYPERSENSITIVITY TO LOW PI-ELICITED PRIMARY ROOT SHORTENING*, 35 genes); *BT1/2* (*Bric-a-Brac/Tramtrack/Broad 1*, seven genes); and *TGA1/4* (*TGACG MOTIF-BINDING FACTOR 1*, 10 genes) (**Figures 1A, C**). For N transport network, the 206 candidates were characterized from five transport/transmembrane families and one TF gene family, including NRT2 (two genes), NPF (147 genes), AMT2 (six genes), CLC (16 genes), SLAC/SLAH (27 genes), and R2R3-MYB (eight genes) families (**Figures 1A, C**). N assimilation network consisted of 56 homologs from four enzyme families and one TF gene family, which include NR (seven genes), NiR (six genes), GS (25 genes), GOGAT (nine genes), and SPL (nine genes) families (**Figures 1B, C**). The 142 common regulatory genes of N uptake and assimilation networks belonged to seven gene families, namely NLP (six genes), TCP (Teosinte-branched1/cycloidea/proliferating cell factor1, 36 genes), LBD (14 genes), SPX (SPX domain-containing proteins, 11 genes), CPK (Calcium-dependent protein kinase, 32 genes), CIPK (35 genes), and CBL (Calcineurin B-like protein, eight genes) families (**Figure 1C**).

Subcellular localization analysis showed that structural proteins in N uptake and transport networks were mainly located in the cell membrane or vacuole and those in N assimilation network were mainly distributed in plastids, while the regulatory proteins in these three networks were commonly located in the nucleus (**Table S1**), indicating the structural proteins in these three networks of the N utilization pathway may play a complementary role in N utilization at the cellular level. The subcellular localization of structural proteins for N uptake and transport networks in membrane or vacuole reflected their function in N transportion. In contrast, the structural proteins of N assimilation network were mainly localized in the plastids, suggesting their roles in N redox process.

### Chromosome localization, duplications and evolution of N utilization pathway in *B. napus*

**Figure 2:**
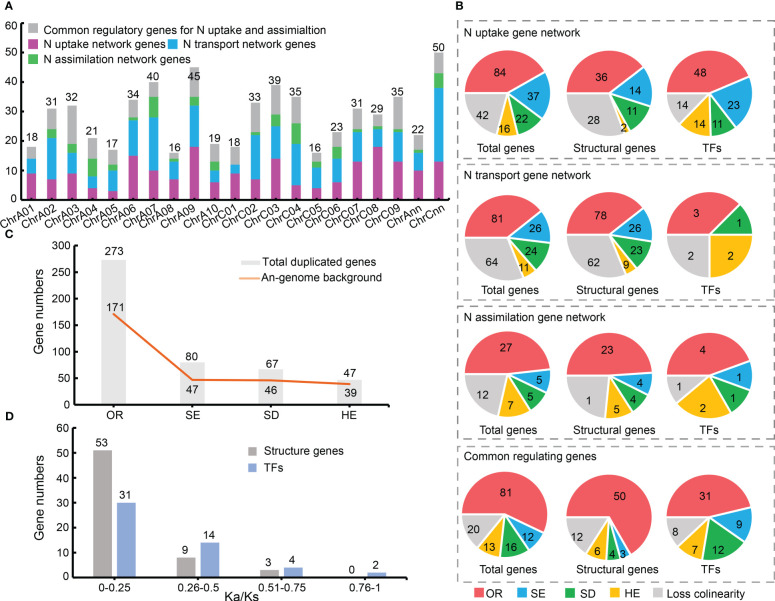
Chromosomal localization and syntenic relationship of nitrogen **(N)** utilization pathway related genes. **(A)** Chromosomal localization of 605 N uptake, transport, and assimilation network genes. Different colors represent three major gene networks and their common regulatory genes. **(B)** Syntenic relationships between 467 N utilization related genes. **(C)** N utilization related genes in an A_n_-sub-genome background. Y-axis represents gene numbers, with A_n_-sub-genome background, and X-axis represents duplication events. **(D)** Selective pressure of N utilization related genes. OR, allopolyploid event; SE, segmental exchange; SD, segmental duplication; HE, homologous exchange.

The distribution trends of candidate N utilization pathway genes in A_n_- (295 genes) and C_n_ (309 genes)-sub-genomes were similar (**Figure S1**, **Table S1**). But the number of candidates on each chromosome was uneven (**Figure 2A**).

Collinearity analysis showed that 467 of the 605 genes had a syntenic relationship in at least one of the three genomes (*Brassica oleracea*, *Brassica rapa* and *B. napus*), while the rest (138) had no syntenic relationship (**Table S3**). Among the 467 genes, 273 (~58.46%) were directly inherited from *B. rapa* (171; ~36.62%) and *B. oleracea* (102; ~21.84%) *via* the allopolyploid event (**Figures 2B, C**), indicating the allopolyploidy was the main driving force for gene expansion of the N utilization pathway in *B. napus*. Meanwhile, small-scale duplication events, including segmental exchange (SE; 80 genes; ~17.13%), segmental duplication (SD; 67 genes; ~14.35%) and homologous exchange (HE; 47 genes; ~10.06%), also played an important role in the gene expansion. However, only four pairs of tandem duplication (TD) genes were found (**Table S3**), indicating its minor role in the gene expansion. Moreover, the three gene networks of N utilization pathway showed similar expansion patterns, with most genes inherited from their ancestors and followed by SE, SD, and HE events respectively (**Figure 2B**). And the TFs in the three networks tended to expand in *B. napus* (**Figure 2B**).

Notably, 58.75% (47 genes) of the SE events of N utilization pathway genes were replaced by A_n_ to C_n_ sub-genome, and ~68.66% genes (46 genes) in this pathway that were inherited from *B. rapa* were subsequently duplicated by SD events in *B. napus*, whereas ~82.98% genes (39 genes) that were inherited from the A_n_-sub-genome were replaced by homologs from the C_n_-sub-genome *via* HE events (**Figure 2C**). The fact that more genes were inherited from *B. rapa* (171; ~62.63%) than from *B. oleracea*, and that more genes from *B. oleracea* genome were replaced by homologs from *B. rapa* genome, indicated the increasing importance of the *B. rapa* genome background in *B. napus* (**Figure 2C**).

A total of 116 duplication gene pairs were identified in *B. napus* (including SD, HE and TD events) by collinearity analysis, including 65 pairs of structural genes and 51 pairs of TFs (three genes with serious sequence deletion in the coding regions were excluded for technical reason). Selective pressure analyses showed that all 116 duplication pairs had Ka/Ks < 1 (**Table S4**), indicating they were under purifying selection or neutral selection (**Figure 2D**). The Ka/Ks values of most structural genes in the N utilization pathway were markedly lower than those of the TFs, suggesting that structural genes had undergone greater purifying selection pressure. These results revealed that purifying selection maybe the main evolutionary power acting on the N utilization pathway.

### Potentail transcriptional regulation profile of N utilization pathway in *B. napus*

**Figure 3:**
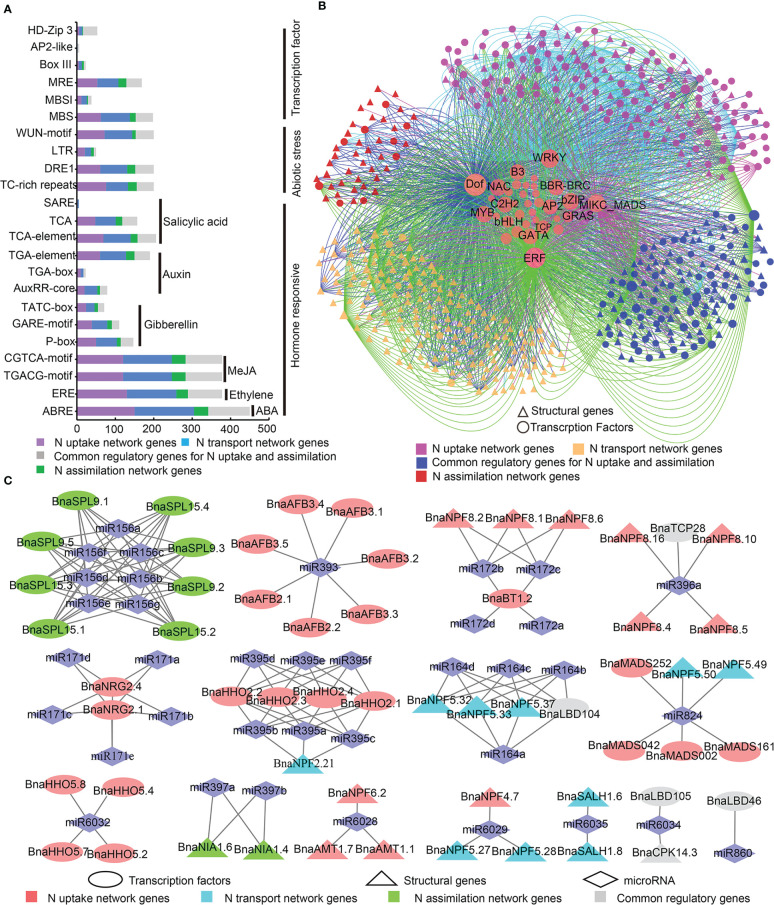
Transcriptional and post-transcriptional analysis of N utilization related genes. **(A)** Twenty-three important *CRE* types in the promoters of N utilization related genes. The X-axis represents gene numbers. **(B)** Potential regulatory interaction between transcription factor and N utilization related genes. **(C)** Potential miRNA regulatory relationships between N utilization related genes.

Up to 127 types of 69,424 *cis*-acting regulatory elements (*CREs*) were identified in the promoter regions (-1,500 bp) of the 605 N utilization network genes (**Table S5**). A majority of these were core *cis*-elements (e.g., CAAT-box and TATA-box) and light responsive *cis*-elements. Meanwhile, many hormone *cis*-responsive elements, abiotic stress responsive *cis*-elements, and TF binding sites were found in the promoter regions of the candidates (**Figure 3A**). The abscisic acid (ABA) (ABRE; 450/605 genes), ethylene (ERE; 378 genes), and MeJA (Methyl jasmonate) (TGACG-motif and CGTCA-motif, 378 genes) responsive *cis*-elements were the significantly enriched *CREs* in the promoters of most N utilization genes (**Figure 3A**), suggesting the potential hormone inducing expression pattern of the N utilization network. Four types of abiotic stress responsive *cis*-elements, namely stress (TC-rich repeats; 200 genes), low temperature and salt stress (DRE1; 50 genes), low temperature (LTR; 200 genes) and wound (WUN-motif, wound-responsive element, 200 genes) responsive *cis*-elements, were detected in many promoter regions of N utilization genes (**Figure 3A**). Four major types of potential TF binding sites, including a MYB binding site (MBS, 198 genes; MBSI, 38 genes; MRE, 169 genes), a HD-Zip3 binding site (HD-Zip 3, 34 genes), a Box III binding site (23 genes), and an AP2-like binding site (six genes), were also identified (**Figure 3A**).

Based on the PlantTFDB database, 4,237 potential regulatory interactions between TFs (481 genes from 35 gene families) and N utilization pathway genes in *B. napus* were predicted (**Figure 3B**). Among these, members of four TF families, Dof (DNA binding with one finger), WRKY (Tryptophan-arginine-lysine-tyrosine), MIKC_MADS (MIKC-type MCM1-AGAMOUS-DEFICIENS-SRF-box), and ERF (Ethylene Responsive Factor), exerted wide and preferential regulatory effects on N utilization network genes. The Dof gene family members (15 genes) may regulate genes in all three major networks of the N utilization pathway; WRKY family members (36 genes) regulate many genes in the N uptake network; MIKC_MADS family members (18 genes) mainly regulate genes in N uptake and common regulatory genes in N uptake and transport networks; ERF family members (72 genes) mainly regulate genes in N transport and common regulatory genes in N uptake and transport networks. Other types of TF genes, such as members of GRAS (derived from GAI, RGA and SCR), bHLH (basic helix-loop-helix), and bZIP families may play potential regulatory roles in the N utilization pathway.

MicroRNAs (miRNAs) played an important role in gene expression. Thus, we predicted potential miRNA targets in the CDS sequences of the 605 N utilization genes *via* psRNATarget online software. In all, we detected 156 pairs of regulatory relationships among 37 types of miRNAs (**Figure 3C**, **Table S6**), including 67 pairs (~43.95%), 21 (~13.46%), 60 (~38.46%), and 8 (~5.13%) among the N uptake network, N transport network, N assimilation network, and common regulatory genes of N uptake and transport networks, respectively. These results revealed the potential regulatory effects of miRNAs on N utilization-related genes.

### Low-nitrogen (LN) RNA-seq in *B. napus* and expression analyses of N utilization pathway

**Figure 4:**
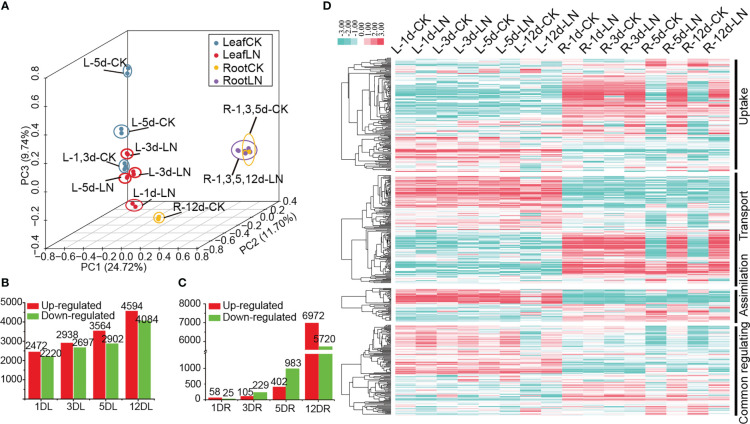
Transcriptome analysis of *B. napus* leaves and roots under low-nitrogen (LN) stress and LN stress expression profile of N utilization network. **(A)** Principal component analysis (PCA) based on expression profile. **(B)** Differentially expressed gene (DEG) numbers in roots. **(C)** Differentially expressed gene (DEG) numbers in leaves. Red indicates upregulated genes, while green indicates downregulated genes. **(D)** LN stress expression profile of the N utilization pathway. “L” represents leaves; “R” represents roots; “d” indicates the day after LN treatment; “CK” indicates Control check. Dataset containing candidate details is provided in **Table S10**.

We constructed a high-quality RNA-seq dataset of the leaves and roots of *B. napus* ZS11 ecotype at five-leaves stage subjected to LN treatment for 1, 3, 5, and 12 d (PRJNA612634) (**Figure S2A**, **Table S7**). In all, 72,830 genes were mapped, including 3,645 new detected transcripts (**Table S8**). Principal component analysis (PCA) separated leaf and root samples into two groups, suggesting that these two organs exhibited different expression patterns under LN treatment (**Figure 4A**).

Although no obvious phenotypic differences between the plants of those treatments, a total of 25,864 non-redundant differentially expressed genes (DEGs) were identified in leaves (17,986 DEGs) and roots (12,900 DEGs), which were divided into six typical expression patterns using K-means clustering method (**Figure S2B**). This suggested that these DEGs were involved in response to LN stress processes in *B. napus*. Genes in Cluster 1 (7,823 genes) and Cluster 5 (4,987 genes) were highly expressed and up-regulated under LN stress on day 12 in roots and leaves, respectively; Genes in Cluster 3 (2,873 genes) were expressed in leaves and up-regulated under LN stress on day 12; Genes in Cluster 6 (3,112 genes) were up-regulated under LN stress on day 3 and 5 in leaves; while those in Cluster 2 (4,651 genes) and Cluster 4 (2,418 genes) were down-regulated under LN stress on day 12 in roots and leaves, respectively. Additionally, 239 and six DEGs were continuously differentially expressed across all four treatments under LN stress in leaves and roots, respectively (**Figure S2C, D**). In general, the number of DEGs in both roots and leaves increased with processing time; however, the trends in the two organs were quite different (**Figures 4B, C**). The number of DEGs in roots increased gradually from 83 to 1,385 under LN treatments from days 1 to 5, and remarkably increased to 12,692 on day 12, suggesting that the N starvation response was mainly triggered on day 12 (**Figure 4C**); While in the leaves, the number of DEGs increased steadily from 4,692 to 8,679 from days 1 to 12 under LN treatment (**Figure 4B**). Moreover, the DEGs in leaves were significantly enriched in many metabolic processes, such as amino acid biosynthesis, carbon N utilization, and photosynthesis (**Figures S2E**, **S3A**), whereas those in roots were mainly enriched in amino acids biosynthesis, plant hormone signal transduction, starch and sucrose metabolism, and N utilization (**Figures S2F**, **S3B**). Overall, our results showed that the LN response mechanism between leaves and roots were different, with the leaves being more sensitive to N deficiency than roots.

Based on the LN RNA-seq dataset (Dataset 1, D1), the stress expression profile of N utilization pathway was investigated. Up to 474 (~78.35%) of the 605 N utilization pathway genes showed detectable expression levels (FPKM ≥ 1) in roots and/or leaves, while the remaining showed no or weak (FPKM < 1) expression in the samples (**Figure 4D**, **Table S9**). Moreover, 330 of them were differentially expressed under LN treatment. The expressions of the three N utilization pathway networks were divided into two opposite patterns, which were either preferentially and even specially expressed in roots or leaves, showing a temporal and spatial expression trend (**Figure 4D**). In roots, most genes were differentially expressed after 5 d (261) and 12 d (254) under LN stress treatments, displaying a delayed LN induced expression profile. Meanwhile, some genes were continuously expressed or differentially expressed in leaves or roots, such as *BnaNRT2.3*, *BnaNIA1.7*, and *BnabZIP1a*, suggesting a potential functional characteristic in *B. napus* (**Table S10**). Moreover, 375 out of the 481 TFs that were predicted by PlantTFDB had detectable expression levels in the RNA-seq data, whose expression pattern was divided into two types. Type I (261 genes) showed an inducible expression pattern which was mainly expressed in roots with most genes up- (182 genes) or down-regulated (79 genes) on day 5 and 12 under LN stress; while Type II (114 genes) was mainly expressed in leaves and maintained high expression levels regardless of LN stress (**Figure S4**).

Using the public RNA-seq dataset (PRJNA358784) (Dataset 2, D2), we characterized the spatio-temporal expression profile of the N utilization pathway genes in 60 *B. napus* samples across different developmental stages. A total of 527 candidates had detectable expression levels in the tissues investigated (**Figure S5**, **Table S11**). Most of them were preferentially expressed in vegetative organs (e.g., roots, stems, and leaves), while relatively less genes were highly expressed in reproductive organs. In general, most expressed candidates were overlapped in D1 and D2, but 16 genes that were not expressed under normal conditions in D2 displayed detectable expression levels in D1 (**Table S9**), suggesting a specific function in LN stress response. Additionally, we found that ~63.64% of the 118 duplicated pairs mentioned above shared conserved expression patterns in these two datasets (|Pearson correlation coefficient| ≥ 0.6), indicating functional redundancy; while ~36.36% (27) of them exhibited obviously different expression patterns (|Pearson correlation coefficient| < 0.6) (**Table S12**), demonstrating that these duplicated genes had undergone expression divergence and even functional differentiation during evolution.

### Co-expression analysis of N utilization pathway in *B. napus* based on RNA-seq datasets

We performed a co-expression analysis based on the above two RNA-seq datasets (D1 and D2). Co-expression relationship pairs with PCC value ≥ 0.6 (or ≤ -0.6) and p-value ≤ 0.01 were obtained.

In D2, a total of 2,143 co-expression relationship pairs of N utilization pathway were identified, forming a complex gene network that was divided into four major groups (Group I to IV, **Figure S6A**). In Group I (24 genes), almost all (23) were expressed in roots and stems during all developmental stages (FPKM ≥ 1) with many (13) highly expressed in roots (FPKM ≥ 20), which were mainly involved in N uptake and transport networks (**Figure S7**, **Table S13**). In Group II, all 17 genes were mainly involved in N uptake and assimilation process which were preferentially expressed in leaf and silique pericarp tissues, especially highly expressed (FPKM ≥ 20) in leaves (**Figure S7**, **Table S13**). In Group III, all nine genes were highly expressed in flower tissues (pistil and inflorescence tip) and young silique pericarp (**Figure S7**, **Table S13**). In Group IV, all 15 genes were highly expressed in roots, stems, leaves, and early cotyledons, which were associated with N uptake, transport, and assimilation processes (**Figure S7**, **Table S13**). Together, these results indicate that N utilization pathway genes may play roles during the entire growth period of *B. napus* by coordinating with each other in a temporal and spatial pattern.

**Figure 5:**
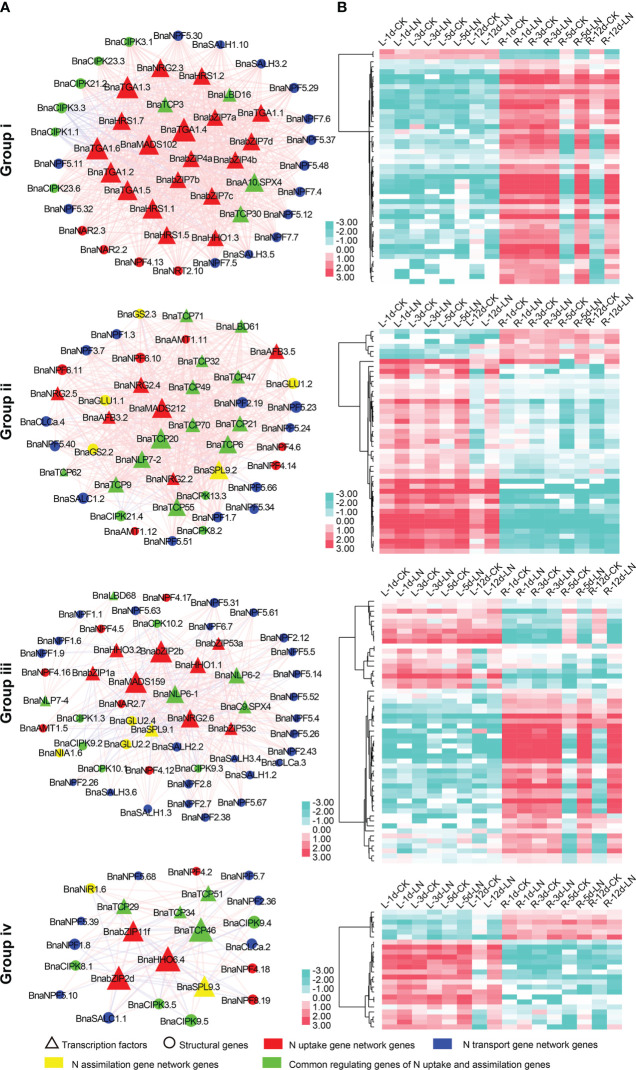
The four major groups (i to iv) of the LN stress co-expression gene network in N utilization pathway. **(A)** depicts the gene network, and **(B)** depicts the corresponding LN stress expression profile of genes in the group. The red lines between two nodes represent a positive correlation, and the gray lines represent a negative correlation. “d” indicates the day after LN treatment; “L” represents leaf sample; “R” represents root sample.

In D1, up to 6,940 co-expression relationship pairs were obtained which were divided into four major groups (i to iv; **Figure S6B**). In Group i, the majority genes (40/47) were specially expressed and markedly upregulated (FC ≥ 2) in roots after 5 and 12 d under LN stress (**Figure 5**, **Table S14**), and 37 (~78.72%) of them were related to N uptake and transport, suggesting a role in the N acquisition process. By contrast, most genes (33/45) in Group ii were specially expressed and upregulated in leaves after 12 d under LN treatment (**Figure 5**, **Table S14**), and all of them were associated with N transport and assimilation processes. In Group iii, the majority genes (49/53) were clustered into two expression patterns with most specially expressed in roots and some in leaves (**Figure 5**, **Table S14**); especially 31 genes related to N transport were specially expressed and upregulated in roots after 5 and 12 d under LN treatment. In Group iv, 12 out of 24 genes were differentially expressed in leaves after 12 d under LN treatment, while 10 were differentially expressed in root after 5 and 12 d under LN stress (**Figure 5**, **Table S14**), and all of the 22 genes were associated with N transport.

Six potential hub genes, *BnaNLP6-1*, *BnaNLP6-2*, *BnaSPL9.1*, *BnaSPL9.2*, *BnaSPL9.3*, and *BnaTCP34*, were screened in the two co-expression gene networks based on D1 and D2 datasets using cytoHubba in the Cytoscape software. Among which, *BnaNLP6-2* was highlighted for high expression level (FPKM > 20) and dramatically differential expression level (FC ≥ 4) in roots under LN stress.

### Expression profiles of N utilization-related genes under LN conditions in *B. napus* roots

On the basis of the DEGs and co-expression analyses of the LN RNA-seq dataset (**Figures 4**, **5**), nine DEGs from NRT2 (*BnaNRT2.10*), NPF (*BnaNPF2.12*, *BnaNPF2.8*, and *BnaNPF5.4*), GARP (*BnaHHO5.1*), NLP (*BnaNLP6.1*), bZIP (*BnabZIP53c*), LBD (*BnaLBD106*) and MYB (*BnaMYB183*) families which represented the structural genes and regulatory genes as well as the hub genes in N utilization pathway were selected to further analyse their potential roles in response to N deprivation condition in *B. napus* by qRT-PCR assay.

**Figure 6:**
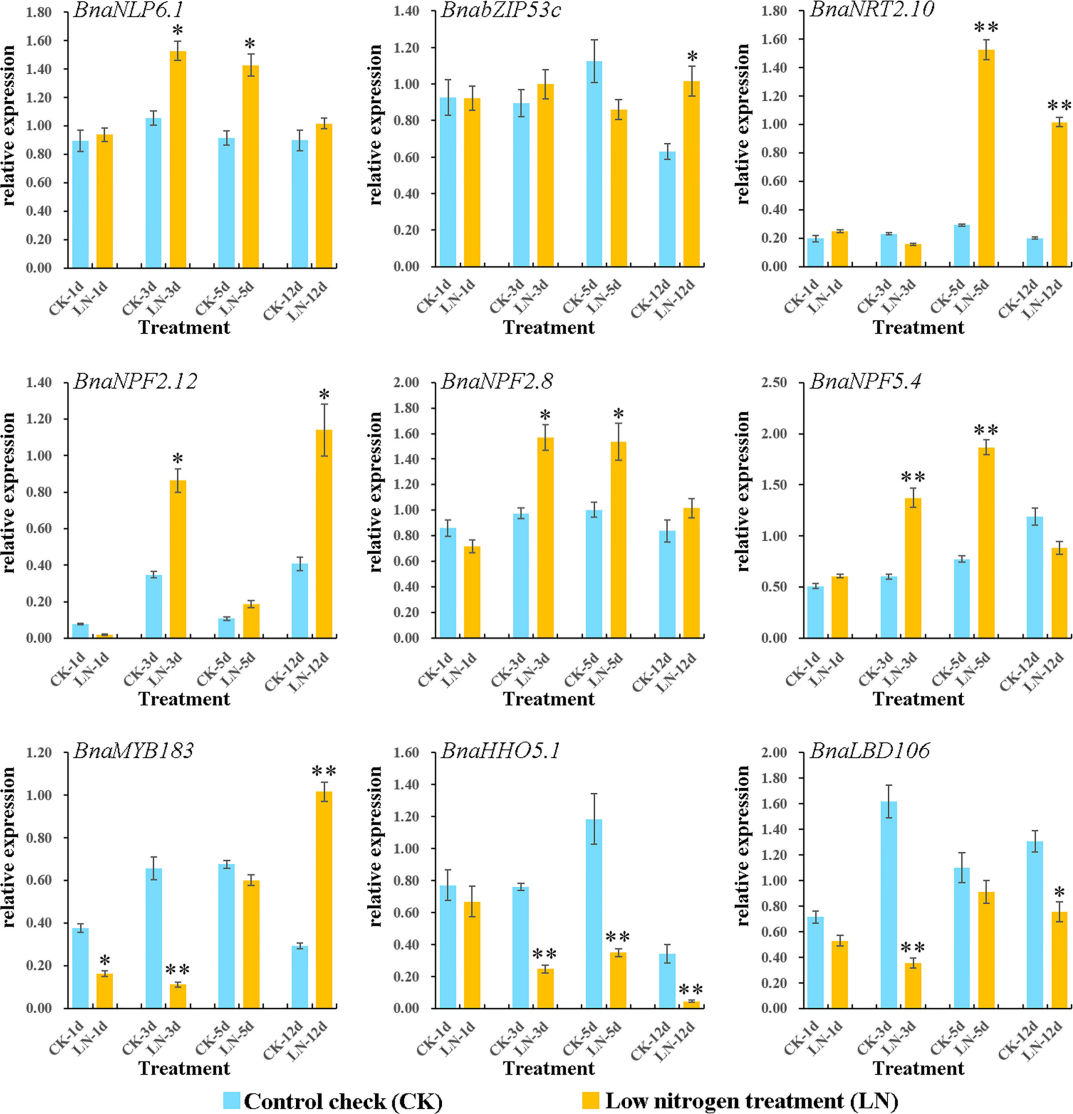
Expressions of nine genes in N utilization pathway under LN treatments by qRT-PCR. The transcript levels were investigated in *B. napus* seedling roots under LN conditions by qRT-PCR method. CK: normal N condition; LN: low N treatment. d: day (s) after LN treatment. Error bars indicate the standard deviation of three independent experiments. “*” indicates significant difference (0.05>p>0.01); “**” indicates extremely significant difference (p<0.01).

As shown in **Figure 6**, similar to those of our RNA-seq analysis, all of the nine genes were evidently induced to varying degrees at different times by LN treatments. Among which, the expression of *BnaNLP6-1* gene was continuously up-regulated in roots under LN stress with significantly higher expression levels on 3 and 5 days, compared with the untreated control. Similarly, *BnaNRT2.10*, *BnaNPF2.12*, *BnaNPF2.8*, *BnabZIP53c*, and *BnaNPF5.4* genes showed an upregulation trend under LN stress conditions, where *BnaNPF2.8* and *BnaNPF5.4* were strongly up-regulated on 3 and 5 days while *BnabZIP53c*, *BnaNRT2.10*, *BnaMYB183*, and *BnaNPF2.12* were commonly up-regulated at the early stage (on 12 day) displaying a delayed LN induced expression profile. Conversely, *BnaHHO5.1* and *BnaLBD106* genes were down-regulated under LN stress treatment. These results demonstrated that the candidates obviously respond to LN stress, which make them the candidates for further study the LN stress resistance mechanism in *B. napus*.

### Distribution and LN stress expression profile analysis of N utilization pathway in land plants

**Figure 7:**
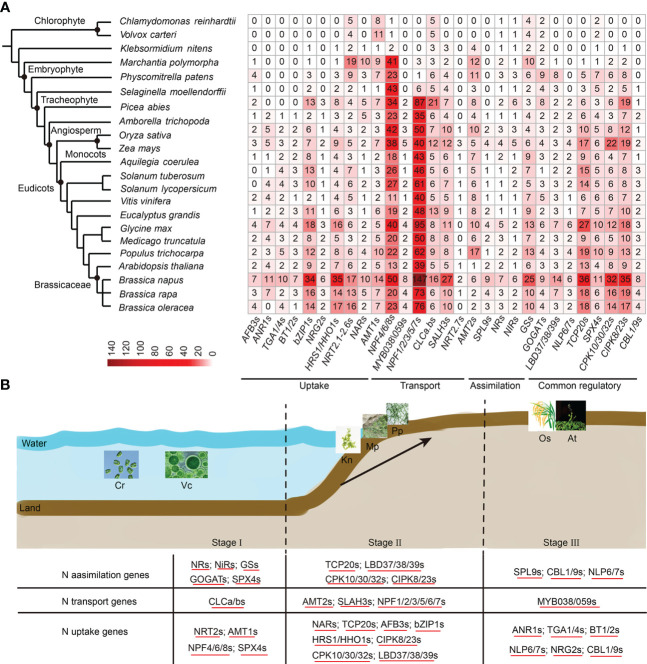
The distribution of N utilization pathway in land plants. **(A)** The distribution of N utilization pathway genes in 22 typical plant species. Homologous genes are marked at the bottom. The number in each square represents the number of genes. The color legend bar has been placed in the lower left corner. **(B)** The evolution and N stress expressions of the N utilization pathway in plants. The evolution trend is divisible into three stages (Stage I, Stage II, and Stage III). Stage I represents the origination of N uptake and the assimilation gene network in Chlorophyta. Stage II represents the process of perfecting N utilization pathways during the evolution from aquatic plants to terrestrial plants. Stage III represents a more complex N utilization pathway driven by increasing transcription factor types and numbers in higher land plants. The homologs across different species that shared similar LN stress expression profiles are underlined. The detailed N stress expression information of N utilization pathway genes is provided in **Figure S8** and **Table S16**.

To evaluate the distribution and expansion trends of N utilization pathway in plants, we broadened our dataset to cover more representative plant lineages (22 species), ranging from aquatic green algae to angiosperms (**Figure 7A**, **Table S15**). A total of 4,267 N utilization-related homologs from the 25 gene families were obtained (**Figure 7A**, **Table S15**). We found that N utilization-related genes were present earlier in Chlorophyta, and showed a rapid gene expansion trend from aquatic alga to angiosperms. For example, only 29 homologs were encoded in single-celled chlorophytes, *C. reinhardtii*, while 129 homologs were present in moss (*P. patens*) and up to 605 homologs in *B. napus*. Accordingly, the genes in each network of N utilization pathway showed a gradual increase in number and type along with plant evolution, resulting in a more complex gene regulatory network in angiosperms (**Figure 7A**). Overall, the evolution and expansion of this pathway in plants may be divided into three major stages (**Figure 7B**). The first stage representing the origin of the main structural genes (nine of the 29 types of homologs) occurred at least in Chlorophyta. At this stage, in contrast to N transport network, the primary structural gene networks of N uptake and assimilation had already originated. The second stage represents the establishment of the main regulatory genes in N utilization pathway at the very beginning of the transition from aquatic to terrestrial plants. At this stage, the majority of structural genes related to N utilization, especially those involved in N transport, and six out of the 16 TF families, had emerged in basal land plants. Thus far, the N utilization system had become more precise, consistent with the morphology and structural complexity of land plants evolved from aquatic to terrestrial forms. The extensive gene expansion of this pathway appears to have initially occurred in Embryophyta, as the number of some genes (e.g., *NPF4/6/8s* and *NRT2s*) were significantly increased in *M. polymorpha*. The third stage represents perfection of N utilization pathway early in basal angiosperm, *A. trichopoda*. The types, instead of the number, of structural genes in N utilization pathway were highly conserved in angiosperms during evolution; by contrast, the types and number of the regulatory genes were still increasing, and the homologs of many regulatory genes (e.g., *AtANR1, AtTGA1/4,* and *AtNLP6/7*) emerged first, leading to a more complex gene network in angiosperms, suggesting that regulatory genes are the factors underlying LN tolerance in different plants/crops. The wide distribution and expansion of N utilization pathway along with plant evolution further support the reliability of the large number of homologs and complex gene regulatory network found in *B. napus* in this study.

To examine the N stress expression profiles of N utilization pathway genes in other plants, the publicly available RNA-seq datasets of *Arabidopsis, S. tuberosum, S. lycopersicum, O. sativa* and *Z*. mays under N stress treatments were applied. Consistent with *B. napus*, the majority of N utilization pathway genes in these five species were strongly induced in root and/or shoot tissues under LN stress (**Figure S8**, **Table S16**). In rice, 89 of the 151 (59%) genes (with FPKM ≥ 1) were differentially expressed in roots or leaves under LN or HN stress (**Figure S8A**). In maize, all of the 144 genes (FPKM ≥ 1) were differentially expressed in leaves between LN and HN treatments (**Figure S8B**). In tomato, more than 70% of the genes (FPKM ≥ 1) were differentially expressed in roots and/or leaves in both of the two genotypes GO and GU under LN stress (**Figure S8C**). In potato, 62% of the 123 genes (FPKM ≥ 1) were differentially expressed in root, shoot, and/or stolon tissues under LN treatment (**Figure S8D**). In *Arabidopsis*, nearly all of the 160 genes (FPKM ≥ 1) showed a differentially expressed profile in roots and/or leaves in response to varying N supply (**Figure S8E**). Moreover, the expression patterns of N utilization pathway gene networks differed remarkable between root and leaf samples in these species, supporting the different expression spectrums of the two organs in response to LN stress in *B. napus*. In most cases, the genes were up-regulated under LN condition; however, many genes were downregulated as well. The homologs generally showed conserved LN stress expression pattens across different species. These results indicate that the N utilization pathway genes were highly and commonly sensitive to N stress response in plants.

Together, our results confirmed that the N utilization pathway was widely distributed throughout the plant kingdom, ranging from aquatic algae to angiosperms, with a rapid expansion trend during plant evolution. The homologs in the N utilization pathway, especially the TFs, were exposed to major radiation during the evolution of land plants, as substantiated by the evolutionary trends of morphological and genome complexity of land plants. And the genes in this pathway showed strong induced N stress expression profiles in many plants, indicating a possible function of this pathway in N stress response.

## Discussion

### LN stress response in *B. napus*

LN stress response mechanisms in many plants, especially crops, have been systematically analyzed *via* the transcriptome with the advent of high throughput sequencing technology (Curci et al., 2017; Sinha et al., 2018; Li et al., 2020). In this study, we constructed a transcriptomic dataset of *B. napus* root and leaf tissues under LN stress at the five-leaf stage.

We found that the number of DEGs in leaves was much higher than that in roots (**Figures 4C, D**), suggesting that the mechanisms underlying the response of these two organs to N starvation varied. Similar results were observed in watermelon (Nawaz et al., 2018). However, in rice, more DEGs were found in roots than the shoots after a 15 d LN stress (Sinha et al., 2018), and more transcriptomic changes were observed in roots than in shoots after long-term LN stress in wheat (Curci et al., 2017) as well. These data suggest that genotype and even the developmental stage may be important factors in LN stress response. Meanwhile, the difference between leaves and roots may be also attributed to treatment time, with leaves being more sensitive to short-term LN stress while roots to long-term LN stress. This may be attributed to the existence of N in leaf vacuoles, as compared to roots, which temporarily provide N nutrients for corresponding biochemical processes of leaves. For instance, in physic nut (*Jatropha curcas* L.), more DEGs were observed in leaves than in roots after 72 h, and in roots than in leaves after 2 d under LN stress (Kuang et al., 2017). Similar results were reported in watermelon and rice (Nawaz et al., 2018; Sinha et al., 2018). In *B. napus*, in contrast to the sharp increasing trend in roots (especially on day 12), the number of DEGs in leaves increased gradually with LN stress treatment time, suggesting that long-term LN stress led to a strong response by roots. Thus, we speculated that the LN response mechanisms that are similar in different plants are influenced by development stages, organs, and LN stress treatment times, etc.

**Figure 8:**
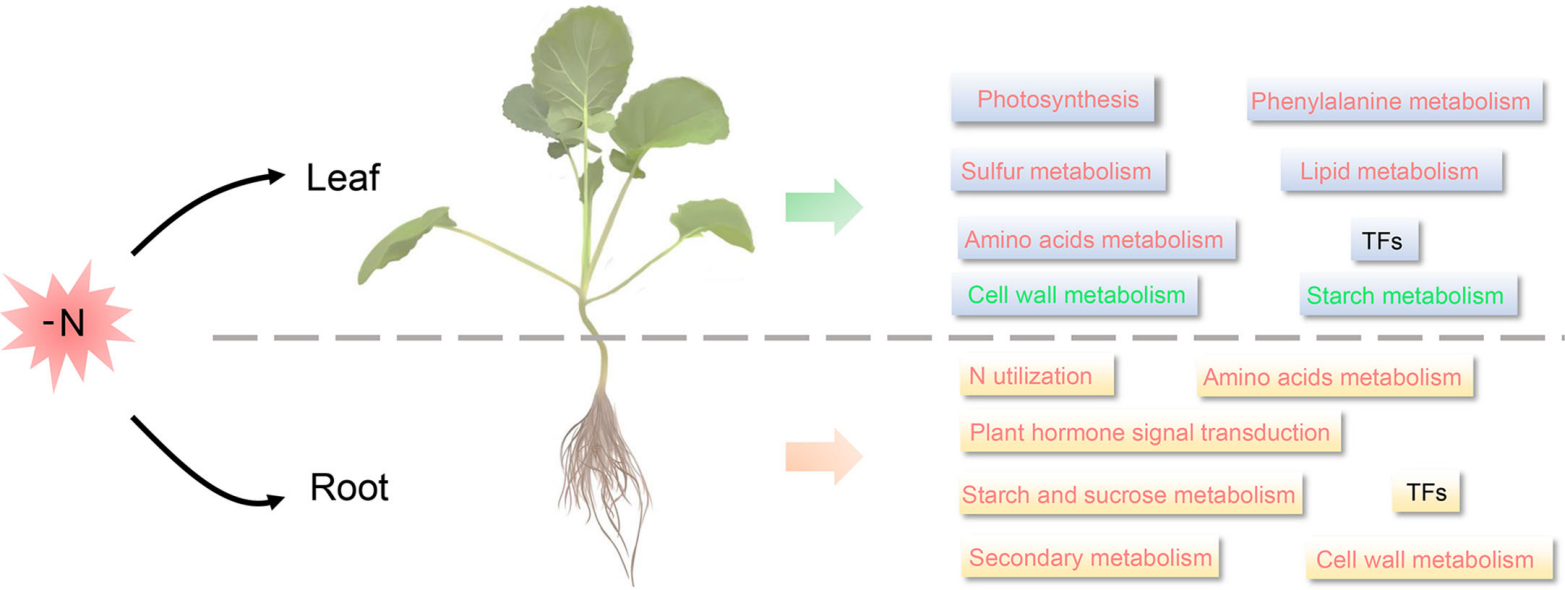
The speculated LN response mechanism in *B. napus* seedling roots and leaves. Red font indicates up-regulation (Fold Change, FC ≥2) under long-term LN stress, green font indicates down-regulation (FC≥2) under long-term LN stress, and black font indicates differential expression under long-term LN stress.

In *Arabidopsis*, under N starvation, sulfur metabolism, amino acid metabolism, TCA cycle and hormone metabolism were significantly upregulated in roots, while photosynthesis, N metabolism, amino acid metabolism, cell wall, and lipid metabolism and carbohydrate metabolism were downregulated in shoots (Krapp et al., 2011). In maize leaves, nitrate reduction, amino acid assimilation, and carbon assimilation-related genes were downregulated in response to LN stress, whereas lipid metabolism, amino acid metabolism and transport, and hormone metabolism were upregulated in roots (Schlüter et al., 2012; Mascia et al., 2019). Similar metabolic responses to LN stress, such as hormone metabolism in roots, and photosynthetic metabolism in shoots, have been reported in many plants, such as rice (Sinha et al., 2018), soybean (Hao et al., 2011), wheat (Curci et al., 2017; Liu et al., 2020), and even green algae (*Myriophyllum aquaticum*) (Wang et al., 2019). These suggested that the mechanisms underlying responses to N deficiency by plants were extremely similar. Similarly, in this study, we found that DEGs in *B. napus* leaves were mainly enriched in photosynthesis, amino acid metabolism, carbon utilization, phenylalanine metabolism, cell wall metabolism, secondary metabolism, and lipid metabolism (**Figure 8**, **Figures S2E**, **S3A**). Meanwhile, the metabolic response to LN stress in *B. napus* roots was very similar to that of many other species as well, such as *Arabidopsis* (Krapp et al., 2011), poplar (Qu et al., 2016), maize (Mascia et al., 2019), rice (Xin et al., 2019a), barley (Quan et al., 2019), and physic nut (*Jatropha curcas* L.) (Kuang et al., 2017). In *B. napus* roots, the DEGs were mainly enriched in amino acids biosynthesis, plant hormone signal transduction, starch and sucrose metabolism, N utilization pathway, cell wall metabolism, lipid metabolism and secondary metabolism (**Figure 8**, **Figures S2F**, **S3B**).

Overall, the metabolic response of roots and leaves to LN stress was different, but it was similar in the same organ across plants. Commonly, genes participated in resistance to N deficiency *via* various photosynthesis, amino acid metabolism, lipid metabolism, energy metabolism, and signal transduction-associated pathways. This provides an interesting research topic on the spatio-temporal LN stress response patterns in a broader array of plant taxa, which will undoubtedly improve our understanding of the mechanisms underlying the response of the two organs to N starvation.

### Potential roles of N utilization network genes in *B. napus*

Starting in as early as 1988, the molecular basis of N utilization has been studied *via* N assimilation genes (Cheng et al., 1988). Subsequently, many studies focused on screening the functional genes involved in N utilization in the past three decades, leading to the discovery of numerous N-associated genes in many plant species (Almagro et al., 2008; Lin et al., 2008; Krapp et al., 2014; Wang et al., 2018). For instance, *Arabidopsis NRT2.1*/*2.2*/*2.4* and rice *OsNRT2.3a*/*OsNRT2.4* genes were demonstrated to involve in high-affinity nitrate uptake/transport processes (Cerezo et al., 2001; Filleur et al., 2001; Tang et al., 2012; Chen et al., 2020); *Arabidopsis AMT1;2* and rice *OsAMT1;1* genes acted as high-affinity NH4(+) transporters (Yuan et al., 2007; Ranathunge et al., 2014); homologs of *NIA1/2*, *NiR1*, *GS2*, and *GLU1* genes played an important role in N assimilation (Bi et al., 2007; Wang et al., 2007; Lothier et al., 2011), etc. In general, the expressions and functional characteristics of the N utilization-associated genes were tightly related to N level in the environment (Bi et al., 2007; Wang et al., 2007). For example, in *Arabidopsis*, *NRT2.1*/*2.2* genes exhibited nitrate-induced expression and were involved in the responses to N availability in the environment at a spatial-temporal manner (Cerezo et al., 2001); *NIA1/2* and *NiR1* genes for nitrate and nitrite reductase respectively, were quickly induced under nitrate treatments (Bi et al., 2007; Wang et al., 2007); *Arabidopsis AMT1;1/1;1;3/2;1* genes were upregulated under N deficiency in roots whose mutants showed a severe growth depression under ammonium supply (Yuan et al., 2007), etc. Similarly, our LN RNA-seq data showed that most homologs of the structural and enzyme genes in N utilization gene networks (~49.28%) were strongly induced under LN conditions in *B. napus* (**Table S10**). For instance, four homologs of *NRT2*, *BnaNRT2.3*, *BnaNRT2.8*, *BnaNRT2.13*, and *BnaNRT2.18*, were upregulated under LN stress (**Figure 4**, **Table S10**). Given gene expression is generally related to its encoded protein function, these results suggested that the homologs of N utilization related genes in *B. napus* also contribute to LN stress response.

Besides, numerous TFs have been widely identified in mediating N utilization process by directly or indirectly regulating expressions of the above mentioned N uptake-, transport- and assimilation-related transporter or enzyme encoding genes (Vidal et al., 2010; Vidal et al., 2015). For example, *bZIP1* mediated rapid nutrient signaling by inducing a large set of genes (e.g., *NRT2.1* and *NIN3*) needed for the N response in *Arabidopsis* in a “hit-and-run” transcription model (Para et al., 2014); *TGA1*/*4* regulated nitrate responses in *Arabidopsis* roots by regulating the expression of nitrate transporter genes *NRT2.1*/*2.2* (Alvarez et al., 2014); *NRG2* played a key role in nitrate signaling regulation through modulating *NRT1.1* expression (Xu et al., 2016); LBD transcription factor genes *LBD37/38/39* acted as negative regulators for nitrate-responsive genes involved in N starvation response (Rubin et al., 2009); etc. Similarly, the present study revealed that diverse candidate TFs (124 genes) in N utilization gene networks showed N-induced expression patterns in *B. napus*, indicating their roles in LN stress response process (**Figure 4D**, **Table S10**). For example, three homologs of *NLP6*/*7* (*BnaNLP6-1*, *BnaNLP6-2*, and *BnaNLP7-4*) and most homologs of *LBD37/38/39* (12/14; ~85.7%) were upregulated in *B. napus* roots under LN stress. Recently, *Arabidopsis MYB59* was proven to play an important role in N utilization process (Du et al., 2019); consistently, the expression of its five homologs in *B. napus* (*Bn2R-MYB045*, *Bn2R-MYB183*, *Bn2R-MYB209*, *Bn2R-MYB255*, and *Bn2R-MYB271*) were significantly upregulated in roots under LN stress, implying a potential function clue in N utilization process. Moreover, some TFs, such as *NLP6*/*7* (Marchive et al., 2013) and *BT1/2* (Araus et al., 2016), acted as a central hub for N utilization gene network. Among them, *NLP6*/*7*, were recognized as master regulators of nitrate signaling and assimilation. They widely modulated the expressions of hundreds of nitrate-inducible genes, including structural genes (e.g., *NRT1.1*, *NRT2.1*/*2.2*) and many N utilization-related TFs (e.g., *LBD37*, *BT1/2*, and *HRS1/HHO1*) by binding to nitrate-responsive *cis*-elements (NREs) in promoter regions (Rubin et al., 2009; Marchive et al., 2013; Medici et al., 2015; Riveras et al., 2015; Liu et al., 2017). Accordingly, we observed that the homologs of these TFs were co-expressed with many N utilization related genes, implying a hub role in *B. napus* as well (**Table S17**). For example, the homolog of *NLP6*/*7*, *BnaNLP6-2* was speculated as a hub gene in the N utilization gene network based on our co-expression analysis (**Figure 5**).

Overall, the LN inducible genes identified in this study constituted a potentially valuable gene source for future gene functional dissection and genetic breeding program in *B. napus*.

## Conclusions

In this study, 605 genes in N utilization pathway were identified from 25 gene families in *B. napus* genome. The allopolyploidy between its ancestors and the small-scale duplication events in *B. napus* are the main driving forces for massive gene expansion of this pathway in *B. napus*. The N utilization-related genes were preferentially expressed in vegetative organs in *B. napus*. A high temporal-resolution transcriptome of *B. napus* leaves and roots under N deficiency was constructed. Most genes in N utilization pathway exhibited strong LN stress induced expression profiles. The LN stress expression characteristics of nine genes were confirmed by qRT-PCR. The N utilization pathway was widely distributed in plant kingdom with a rapid expansion trend. Overall, this study provides important clues about the potential functions of the N utilization-related genes that will be useful for gene function research in the future.

## Data availability statement

The datasets presented in this study can be found in online repositories. The names of the repository/repositories and accession number(s) can be found in the article/**Supplementary Material**.

## Author contributions

PL: Data curation, Visualization, Data analysis, Writing – original draft. ZL: Investigation, Resources. RD: Data curation, Software. ZC: Formal analysis, Methodology. JL: Supervision, Project administration. HD: Conceptualization, Data curation, Project administration, Supervision, Writing – review & editing, Funding acquisition. All authors contributed to the article and approved the submitted version.

## Supplementary material

Supplementary Figure 1 Chromosomal location of nitrogen (N) utilization pathway genes in *Brassica napus.* Chromosome positions of the 605 N utilization pathway genes were mapped on 19 chromosomes. The scale of the chromosome is in megabases (Mb). Chromosome number is indicated at the top of each chromosome and each line represents a gene. The distribution of lines on the chromosomes represents the density of N utilization pathway genes on the chromosomes.

Supplementary Figure 2 Comparative transcriptome analysis of *B. napus* leaves and roots under low-nitrogen stress. **(A)** Sample correlation analysis based on expression values. **(B)** Expression patterns of all DEGs under LN stress using K-means clustering method. **(C)** Venn diagram of DEGs in leaves. **(D)** Venn diagram of DEGs in roots. **(E)** Top ten GO term of all DEGs in leaves. **(F)** Top ten GO terms of all DEGs in roots. Green represents cellular component; orange represents molecular function; blue represents biological process.

Supplementary Figure 3 The Kyoto Encyclopedia of Genes and Genomes (KEGG) pathway enrichment of differential expressed genes (DEGs) in roots and leaves of *B. napus*. **(A)** The KEGG pathway enrichment of DEGs in leaves. The most enriched terms are amino acids biosynthesis, carbon N utilization, ribosome, and photosynthesis. **(B)** The KEGG pathway enrichment of DEGs in roots. The most enriched terms are amino acids biosynthesis, plant hormone signal transduction, starch and sucrose metabolism, and N utilization.

Supplementary Figure 4 The expression patterns of transcription factor genes predicted by PlantTFDB. In total, 375 out of the 481 transcription factor genes predicted by PlantTFDB were analyzed based on their expression levels. The expression patterns of the 375 genes were divided into two parts. Genes in Part I were mainly expressed in roots, whereas the genes in Part II were mainly expressed in leaves. “L” represents leaves; “R” represents roots; “d” indicates the day after LN treatment; “CK” indicates Control check.

Supplementary Figure 5 Spatiotemporal expression profile of N utilization pathway. “L” represents leaves; “R” represents roots; “d” indicates the day after LN treatment; “CK” indicates Control check. Dataset containing candidate details is supplied in **Table S11**.

Supplementary Figure 6 The gene co-expression networks of N utilization pathway under normal N and deficient N conditions. **(A, B)** The gene co-expression network of N utilization pathway genes under normal N and N deficiency, respectively. The red line between two nodes represents a positive correlation, while a gray line represents a negative correlation.

Supplementary Figure 7 Major groups in the spatiotemporal co-expression gene network in N utilization pathway. Groups I to IV represent the four major gene groups in N utilization pathway. “a” depicts the gene network, and “b” depicts the corresponding spatiotemporal expression profile of genes within the group. The red lines between two nodes represents a positive correlation, while the gray represents a negative correlation.

Supplementary Figure 8 The expression profile of N utilization network under LN stress in different plant species. **(A–E)** LN stress expression profile of N utilization pathway in *Oryza sativa*, *Zea mays*, *Solanum lycopersicum*, *Solanum tuberosum*, and *Arabidopsis* respectively. “L” represents leaves; “R” represents roots; “d” indicates the day after LN treatment; “CK” indicates Control check. Dataset containing candidate details is provided in **Table S15**.
