# Supplementary figures and images for: An integrated nitrogen utilization gene network and transcriptome analysis reveal candidate genes in response to nitrogen deficiency in Brassica napus
Source: Front Plant Sci. 2023 May 9;14:1187552. doi: 10.3389/fpls.2023.1187552 (PMC10203523; doi:10.3389/fpls.2023.1187552)

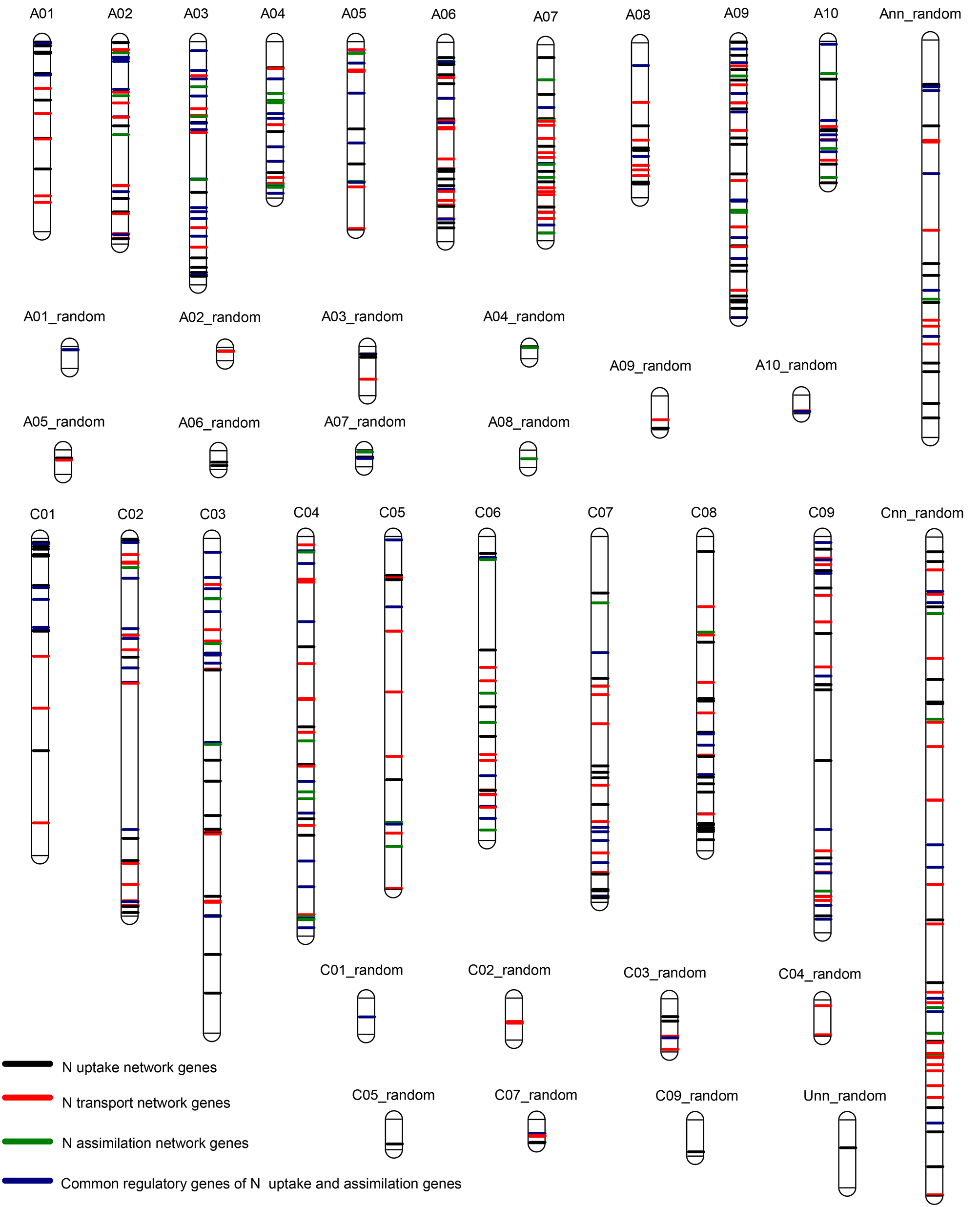

Supplement: Supplementary Figure 1 — Chromosomal location of nitrogen (N) utilization pathway genes in Brassica napus. Chromosome positions of the 605 N utilization pathway genes were mapped on 19 chromosomes. The scale of the chromosome is in megabases (Mb). Chromosome number is indicated at the top of each chromosome and each line represents a gene. The distribution of lines on the chromosomes represents the density of N utilization pathway genes on the chromosomes. [file DataSheet_1.zip › Supplementary data-FTPS/FIGURE S1.tif]

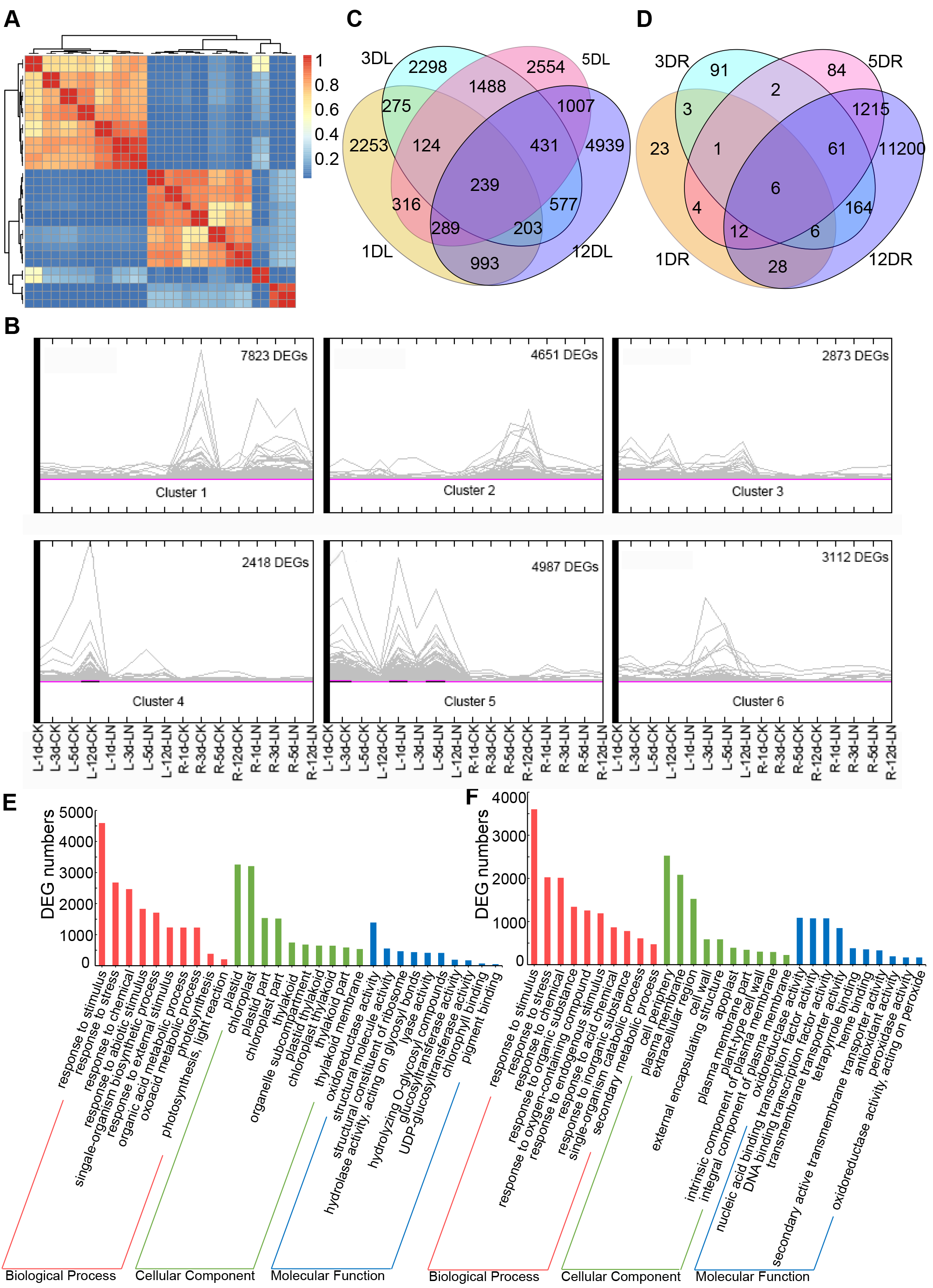

Supplement: Supplementary Figure 1 — Chromosomal location of nitrogen (N) utilization pathway genes in Brassica napus. Chromosome positions of the 605 N utilization pathway genes were mapped on 19 chromosomes. The scale of the chromosome is in megabases (Mb). Chromosome number is indicated at the top of each chromosome and each line represents a gene. The distribution of lines on the chromosomes represents the density of N utilization pathway genes on the chromosomes. [file DataSheet_1.zip › Supplementary data-FTPS/FIGURE S2.tif]

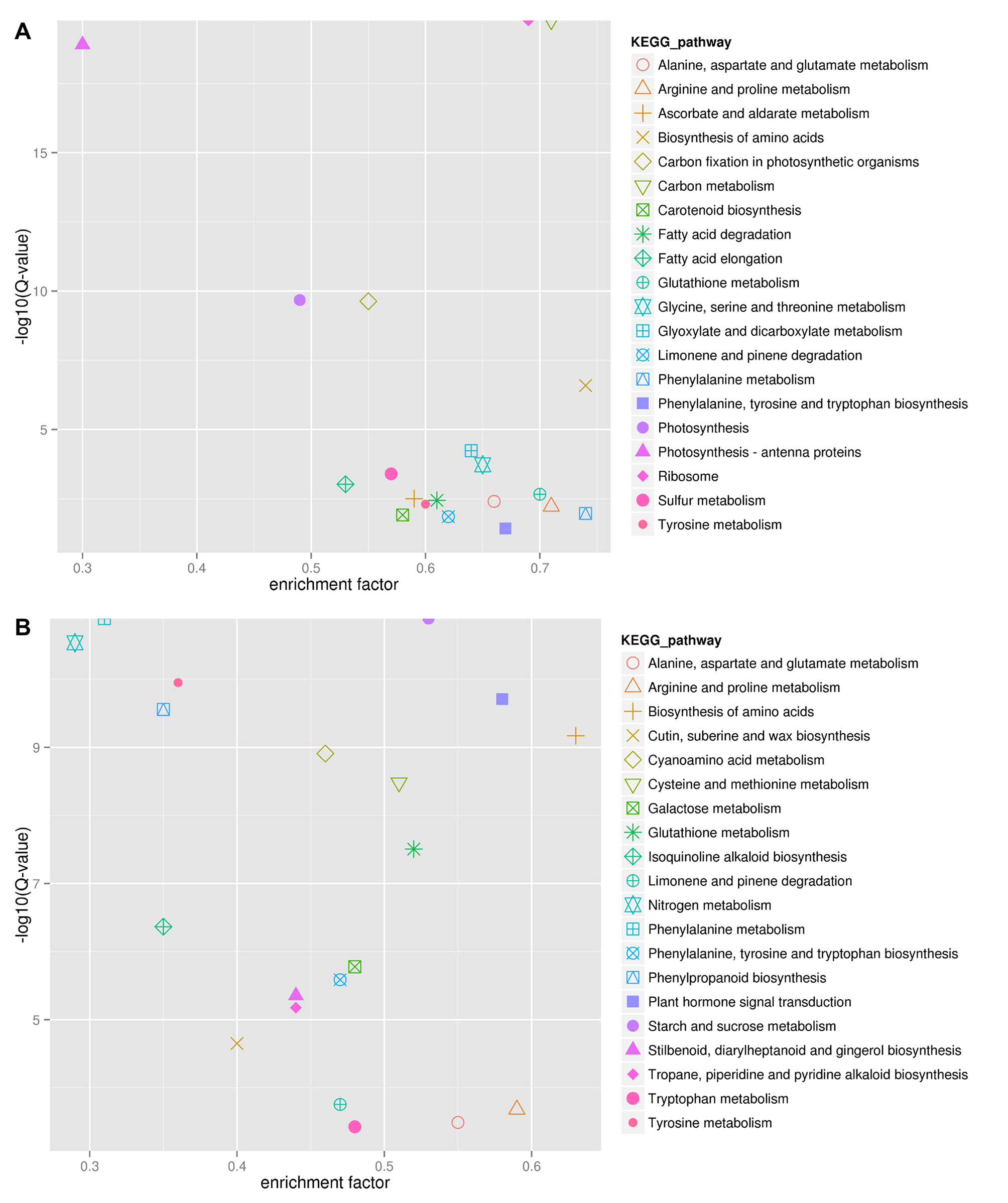

Supplement: Supplementary Figure 1 — Chromosomal location of nitrogen (N) utilization pathway genes in Brassica napus. Chromosome positions of the 605 N utilization pathway genes were mapped on 19 chromosomes. The scale of the chromosome is in megabases (Mb). Chromosome number is indicated at the top of each chromosome and each line represents a gene. The distribution of lines on the chromosomes represents the density of N utilization pathway genes on the chromosomes. [file DataSheet_1.zip › Supplementary data-FTPS/FIGURE S3.tif]

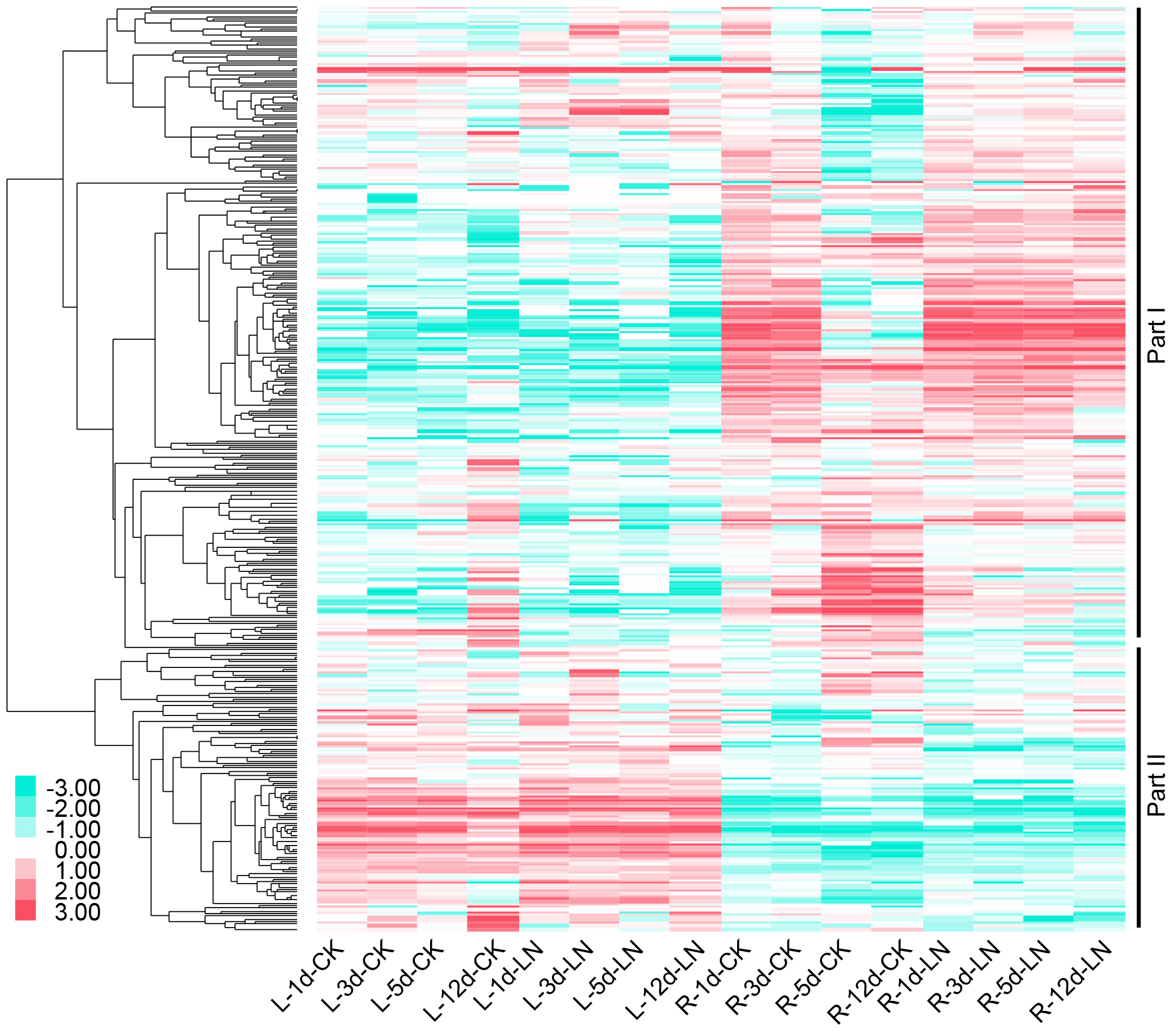

Supplement: Supplementary Figure 1 — Chromosomal location of nitrogen (N) utilization pathway genes in Brassica napus. Chromosome positions of the 605 N utilization pathway genes were mapped on 19 chromosomes. The scale of the chromosome is in megabases (Mb). Chromosome number is indicated at the top of each chromosome and each line represents a gene. The distribution of lines on the chromosomes represents the density of N utilization pathway genes on the chromosomes. [file DataSheet_1.zip › Supplementary data-FTPS/FIGURE S4.tif]

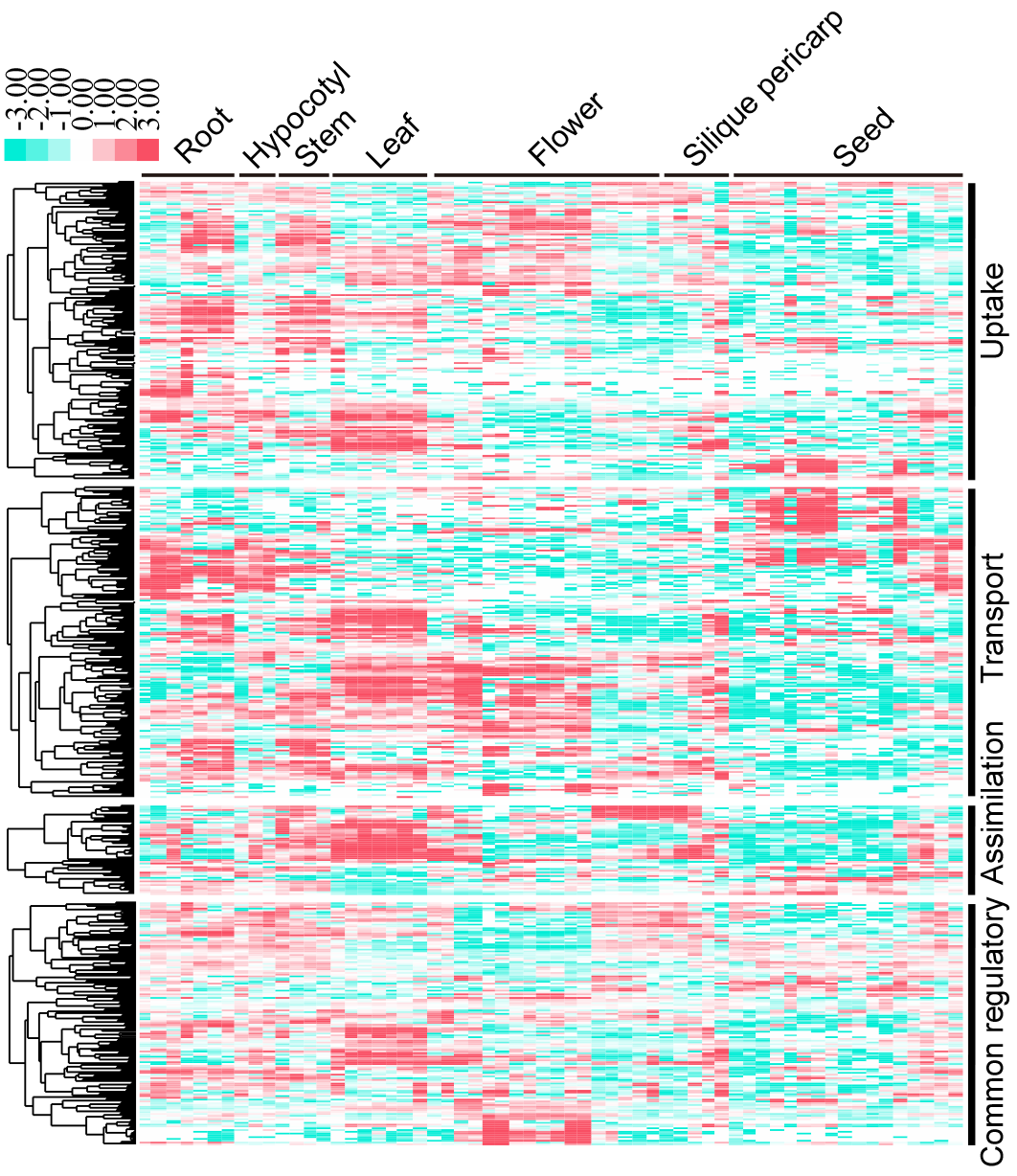

Supplement: Supplementary Figure 1 — Chromosomal location of nitrogen (N) utilization pathway genes in Brassica napus. Chromosome positions of the 605 N utilization pathway genes were mapped on 19 chromosomes. The scale of the chromosome is in megabases (Mb). Chromosome number is indicated at the top of each chromosome and each line represents a gene. The distribution of lines on the chromosomes represents the density of N utilization pathway genes on the chromosomes. [file DataSheet_1.zip › Supplementary data-FTPS/FIGURE S5.tif]

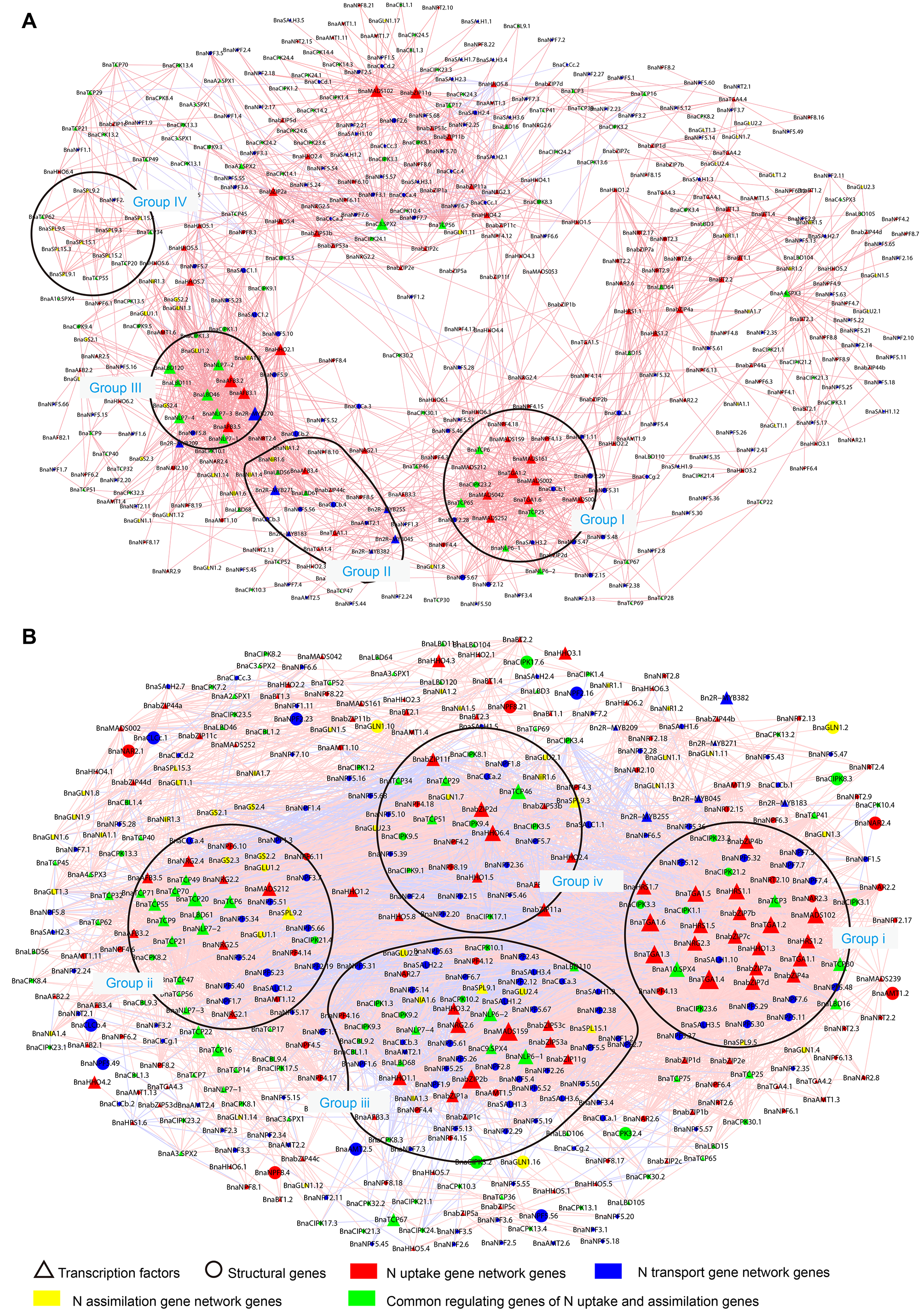

Supplement: Supplementary Figure 1 — Chromosomal location of nitrogen (N) utilization pathway genes in Brassica napus. Chromosome positions of the 605 N utilization pathway genes were mapped on 19 chromosomes. The scale of the chromosome is in megabases (Mb). Chromosome number is indicated at the top of each chromosome and each line represents a gene. The distribution of lines on the chromosomes represents the density of N utilization pathway genes on the chromosomes. [file DataSheet_1.zip › Supplementary data-FTPS/FIGURE S6.tif]

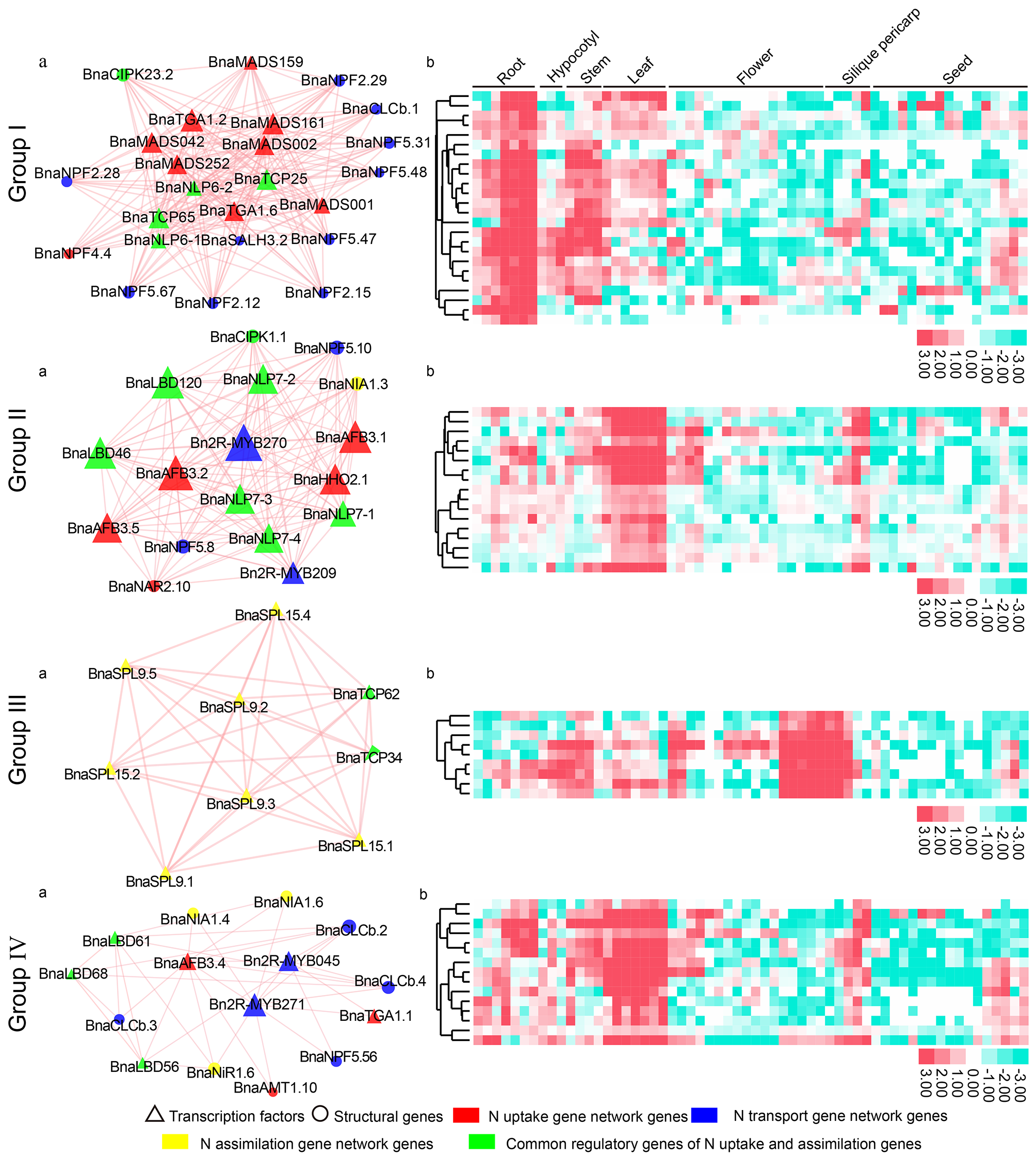

Supplement: Supplementary Figure 1 — Chromosomal location of nitrogen (N) utilization pathway genes in Brassica napus. Chromosome positions of the 605 N utilization pathway genes were mapped on 19 chromosomes. The scale of the chromosome is in megabases (Mb). Chromosome number is indicated at the top of each chromosome and each line represents a gene. The distribution of lines on the chromosomes represents the density of N utilization pathway genes on the chromosomes. [file DataSheet_1.zip › Supplementary data-FTPS/FIGURE S7.tif]

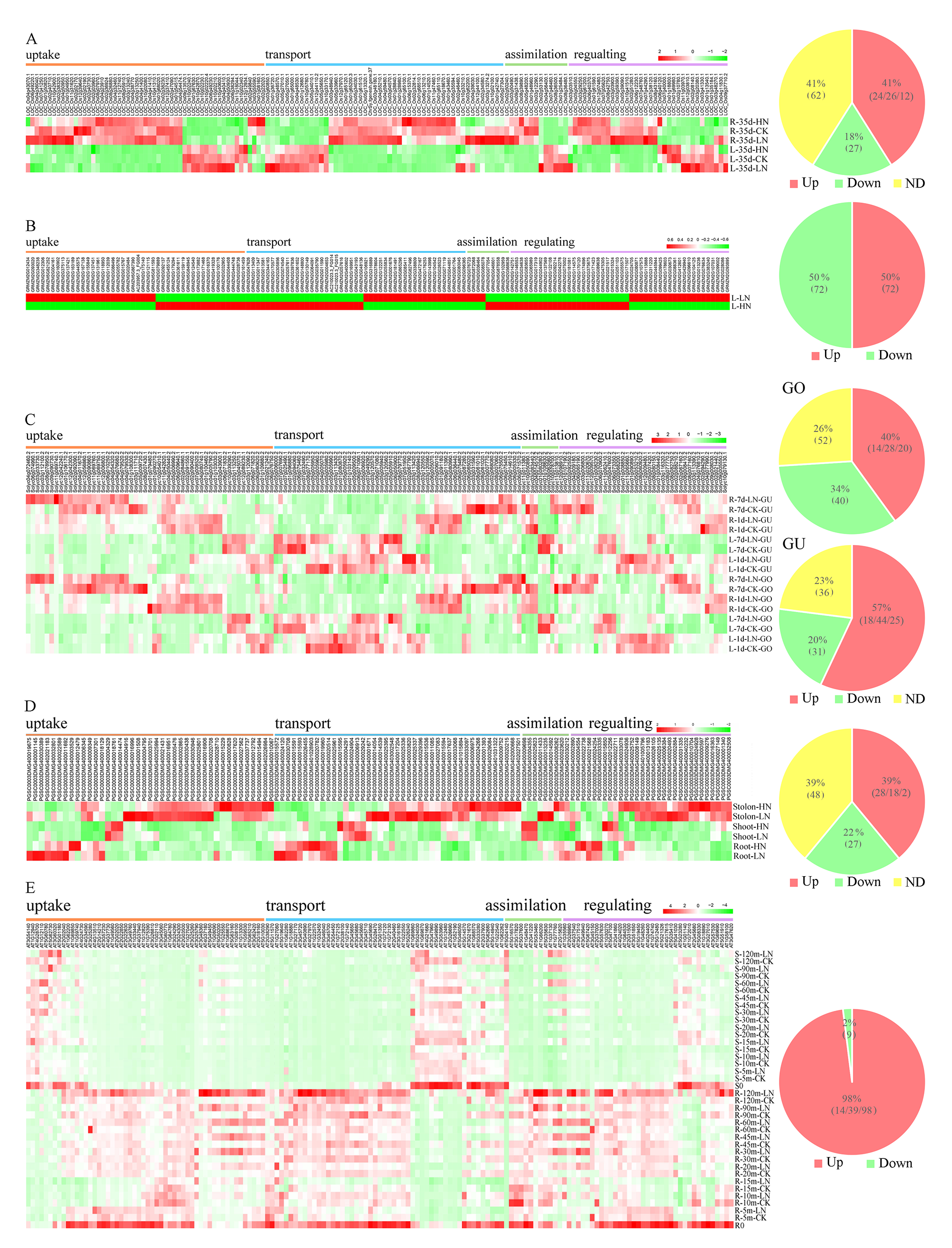

Supplement: Supplementary Figure 1 — Chromosomal location of nitrogen (N) utilization pathway genes in Brassica napus. Chromosome positions of the 605 N utilization pathway genes were mapped on 19 chromosomes. The scale of the chromosome is in megabases (Mb). Chromosome number is indicated at the top of each chromosome and each line represents a gene. The distribution of lines on the chromosomes represents the density of N utilization pathway genes on the chromosomes. [file DataSheet_1.zip › Supplementary data-FTPS/FIGURE S8.tif]
